# Disrupting NtrC function reveals unexpected robustness in a central cell cycle regulatory network

**DOI:** 10.1128/mbio.01962-25

**Published:** 2025-08-18

**Authors:** Hunter North, Molly Hydorn, Jonathan Dworkin, Aretha Fiebig, Sean Crosson

**Affiliations:** 1 Department of Microbiology, Genetics and Immunology, Michigan State University 3078, East Lansing, Michigan, USA; 2 Department of Microbiology and Immunology, Columbia University 5798, New York, New York, USA; Georgia Institute of Technology, Atlanta, Georgia, USA

**Keywords:** bacterial enhancer binding protein, evolution, signal transduction, sigma factor, RNA polymerase, *Caulobacter*

## Abstract

Cell cycle progression relies on coordinated signaling systems that integrate diverse cellular and environmental cues. In *Caulobacter* and related Alphaproteobacteria, the essential sensor histidine kinase CckA initiates a phosphorelay signaling cascade that controls both the transcriptional activity and proteolytic stability of the master cell cycle regulator, CtrA. To uncover regulatory connections between this essential *Caulobacter* signaling pathway and other cellular processes, we selected for mutations that bypassed the loss of CckA function. Among the identified mutations were structurally distinct loss-of-function alleles of the conserved nitrogen assimilation regulator *ntrC*, which differentially suppressed the viability defect of a temperature-sensitive (ts) *cckA* mutant. Complete loss of NtrC function reduces intracellular glutamine, elevates levels of the alarmone ppGpp, and sustains CtrA protein levels, partially rescuing *cckA*(ts) viability. In contrast, loss-of-function NtrC mutants that retain intact AAA+ ATPase and phosphoreceiver domains but lack a DNA-binding domain fully rescue *cckA*(ts) viability and display unexpected transcriptional properties. Specifically, strains expressing these NtrC alleles activate a subset of σ^54^-dependent flagellar genes, even though *Caulobacter* NtrC does not contain the conserved GAFTGA motif required for NtrC-related enhancer-binding proteins to interact with σ^54^-RNA polymerase. We propose that bypass of CckA function by NtrC DNA-binding mutants involves a combination of metabolic changes induced by glutamine limitation and an emergent NtrC activity that modulates transcription from non-native sites on the chromosome. This study reveals unexpected genetic routes to bypass an essential signaling process, highlighting the mutational robustness of a conserved cell cycle control system.

**IMPORTANCE:**

The study of essential genes offers insight into the core biological processes required for life. Yet, gene essentiality is often conditional, shaped by both environmental factors and genomic context. Here, we show that mutations in *Caulobacter ntrC*, a conserved regulator of nitrogen assimilation, enable bypass of an essential signaling process catalyzed by the CckA cell cycle kinase. These mutations coordinately alter metabolism, protein levels, and gene expression in ways that sustain cell growth and division even when the essential regulatory activity of CckA is severely impaired. Our results highlight the capacity of bacterial cells to maintain essential functions through evolutionary reconfiguration of metabolic and regulatory networks.

## INTRODUCTION

Cells have sophisticated molecular mechanisms to monitor both their internal state and the external environment, ensuring the maintenance of homeostasis. In bacteria, a common mechanism that links environmental monitoring to gene expression involves sensor histidine kinase (SHK) proteins, which detect physical and/or chemical cues and regulate adaptive transcriptional responses through phosphoryl transfer to their partner response regulator (RR) proteins (1). SHKs and RRs together form two-component signaling systems (TCSs), one of the most widely conserved gene regulatory mechanisms in bacteria (2). TCSs were initially thought to control gene expression and behavioral responses only under specific environmental conditions (3, 4). However, studies in the years following their discovery uncovered TCSs and multi-component TCS phosphorelays (5) that regulate core cellular processes, including cell envelope biogenesis, cell cycle progression, and cell division. Notably, TCS genes involved in these core functions are often essential for viability under standard cultivation conditions (6–9).

*Caulobacter crescentus* (hereafter, *Caulobacter*) is a metabolically versatile gram-negative bacterium (10–13) that inhabits both soil and aquatic environments (14). A hallmark of its life cycle is asymmetric cell division, which produces two morphologically and functionally distinct daughter cells: a motile swarmer cell and a sessile stalked cell. This developmental asymmetry requires precise spatiotemporal regulation of the cell cycle, which is orchestrated by several TCS regulators. At the center of this TCS regulatory network is the essential response regulator CtrA, whose activation and inactivation governs key transitions in cell cycle progression and cell polarity (15).

**Fig 1:**
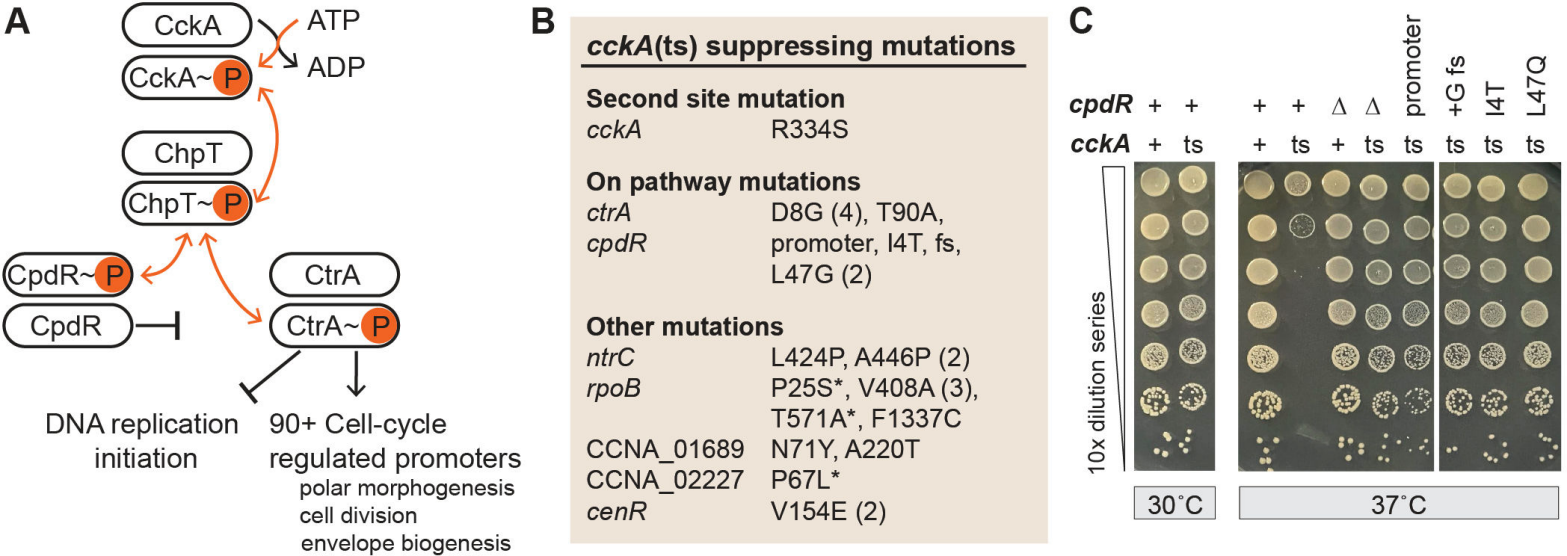
A selection for mutations that bypass the essential function of the cell cycle kinase, CckA, reveals multiple routes of genetic suppression. (**A**) Model of an essential *Caulobacter* cell cycle regulatory phosphorelay. CckA is a bifunctional sensor histidine kinase/phosphatase that coordinates cell cycle progression by regulating the master cell cycle regulator CtrA and its proteolytic adapter CpdR through phosphorylation and dephosphorylation via the intermediary phosphotransferase ChpT. In its kinase state, CckA phosphorylates CtrA (generating CtrA~P), which binds DNA to repress chromosome replication initiation and directly activate transcription of over 90 cell cycle-regulated genes. When acting as a phosphatase, CckA promotes dephosphorylation of CpdR, which licenses CpdR to direct CtrA for degradation by the ClpXP protease. Specific gain-of-function mutations in *ctrA* can bypass the essential requirement for *cckA* (18, 33). To our knowledge, there are no reported conditions under which *ctrA* has been completely deleted. In an otherwise wild-type genetic background, a strain carrying a temperature-sensitive (ts) allele of *cckA* (I484N, P485A) has highly reduced CtrA~P levels and becomes inviable after incubation at 37°C (19). (**B**) Spontaneous suppressing mutations identified in 26 *cckA*(ts) strains that grow at the restrictive temperature (37°C). Highlighted genes were mutated in more than one strain or contained the only polymorphic site in a strain. Table S1 details all mutations identified in each of the 26 strains. Various alleles for each gene are indicated on the right. The number of occurrences is indicated in parentheses for alleles identified more than once. * denotes cases where no other mutations were detected in the strain. fs, frame shift (at codon 80 of 119). CCNA_01689 encodes inosine-5′-monophosphate dehydrogenase; CCNA_02227 encodes a LysR-family transcription factor. (**C**) Serial dilution of *Caulobacter* strains encoding wild-type (+) or mutant alleles of *cckA* and *cpdR* grown for 4 days at the permissive (30°C) or restrictive (37°C) temperature. Temperature-sensitive allele of *cckA*, in-frame deletion of *cpdR* (∆), and *cpdR* mutants recovered in the *cckA*(ts) suppressor selection are indicated.

CtrA is activated via phosphorylation through a multi-component phosphorelay initiated by the essential SHK CckA, which is the sole kinase for CtrA and has both kinase and phosphatase activities (16–19). Phosphorylation of CtrA (CtrA~P) increases its DNA-binding affinity (20), enabling it to directly regulate transcription of over 90 genes involved in DNA replication, cell division, and polar morphogenesis (21) (Fig. 1A). In its phosphatase state, CckA promotes dephosphorylation of both CtrA~P and the single-domain RR CpdR~P. Dephosphorylated CpdR acts as a proteolytic adaptor, targeting CtrA for degradation by the ClpXP protease (17, 22–24) (Fig. 1A), thereby maintaining precise control of CtrA protein levels across the cell cycle. The switch in CckA activity from kinase to phosphatase is regulated by changes in levels of cyclic-di-GMP (25) and ADP (26), and its spatial localization within the membrane (27, 28). Additionally, environmental stress cues are proposed to promote the phosphatase activity of CckA, facilitating CtrA degradation and halting cell division under unfavorable conditions (29). The spatial and temporal dynamics of CckA activity are further refined by additional essential TCS proteins that act in a compartment-specific manner within the *Caulobacter* cell (30–32).

Given the extensive regulatory integration of CckA with multiple essential TCS proteins and its central role in controlling CtrA activity, one might expect that *cckA* would be strictly required for *Caulobacter* viability. However, genetic studies have shown that certain mutations can bypass the requirement for CckA. In particular, gain-of-function mutations in *ctrA* permit complete deletion of *cckA* in *Caulobacter* (18, 33), indicating that structural forms of CtrA can compensate for the loss of its upstream kinase activator. We aimed to identify additional genetic routes that bypass CckA function to gain deeper insight into the architecture of the *Caulobacter* cell cycle control system. To this end, we performed a genetic selection using a strain carrying a temperature-sensitive (ts) allele of *cckA* (19), hereafter referred to as *cckA*(ts). This mutant is not viable at an elevated (i.e., restrictive) temperature due to severely impaired CckA function, which results in a near-complete loss of CtrA~P and a consequent block in cell division (19). Through our selection, we recovered spontaneous suppressing mutations that rescued growth of the *cckA*(ts) mutant at the restrictive temperature. As expected, these included gain-of-function mutations in *ctrA*, as well as *cpdR* mutations that increase CtrA protein stability by interfering with its regulated degradation. We further identified mutations outside of the known cell cycle regulatory network that restored growth of the *cckA*(ts) mutant at the restrictive temperature, including multiple independent mutations in the beta subunit of RNA polymerase (*rpoB*). Additionally, independent mutations in the DNA-binding domain of the nitrogen regulatory protein *ntrC* were isolated, highlighting potential alternative regulatory mechanisms that compensate for the loss of CckA function.

NtrC is a member of a widely conserved class of TCS RRs known as bacterial enhancer-binding proteins (bEBPs) and is best known for its role in activating transcription of genes involved in nitrogen assimilation through its interaction with σ^54^-RNA polymerase (34–36). However, a recent study has shown that *Caulobacter* NtrC lacks a set of amino acids in its AAA+ domain known as the GAFTGA motif (37), which is necessary for interaction with the σ^54^ N-terminal regulatory domain (38). Consistent with this structural limitation, transcriptomic and ChIP-seq analyses indicate that NtrC instead functions at σ⁷⁰-dependent promoters in *Caulobacter*, acting as both a transcriptional activator and repressor (37). In addition to its role in promoting the assimilation of ammonium into glutamine, *Caulobacter* NtrC influences polar stalk development, cell envelope polysaccharide biosynthesis, and binds to numerous sites across the *Caulobacter* chromosome, often overlapping with binding sites for the nucleoid-associated protein GapR and the H-NS-like cell cycle regulator MucR1 (37). These results suggest that NtrC plays a broader role in coordinating nitrogen metabolism with cell cycle and other developmental processes.

Our discovery of *ntrC* mutations that rescue growth of a non-viable *cckA* loss-of-function mutant is congruent with a model in which NtrC can influence cell cycle and cell development. Through a detailed analysis of the genetic interactions between *ntrC* and *cckA*, we identified a complex pattern of genetic suppression, in which distinct loss-of-function mutations in *ntrC* variably rescue the temperature-sensitive defects of a *cckA*(ts) mutant. Our data support a tiered genetic suppression mechanism in which specific *ntrC* loss-of-function alleles enhance ppGpp production, thereby sustaining total CtrA protein levels in the cell, while also supporting transcriptional rescue of dysregulated cell cycle and cell development genes from non-native chromosomal sites. These results illuminate the plasticity of an essential *Caulobacter* signaling pathway and underscore the complex interplay between the noncanonical bEBP NtrC, cell cycle regulation, and cellular development.

## RESULTS

### A selection to identify mutations that suppress lethality of *cckA*(ts)

**Fig 2:**
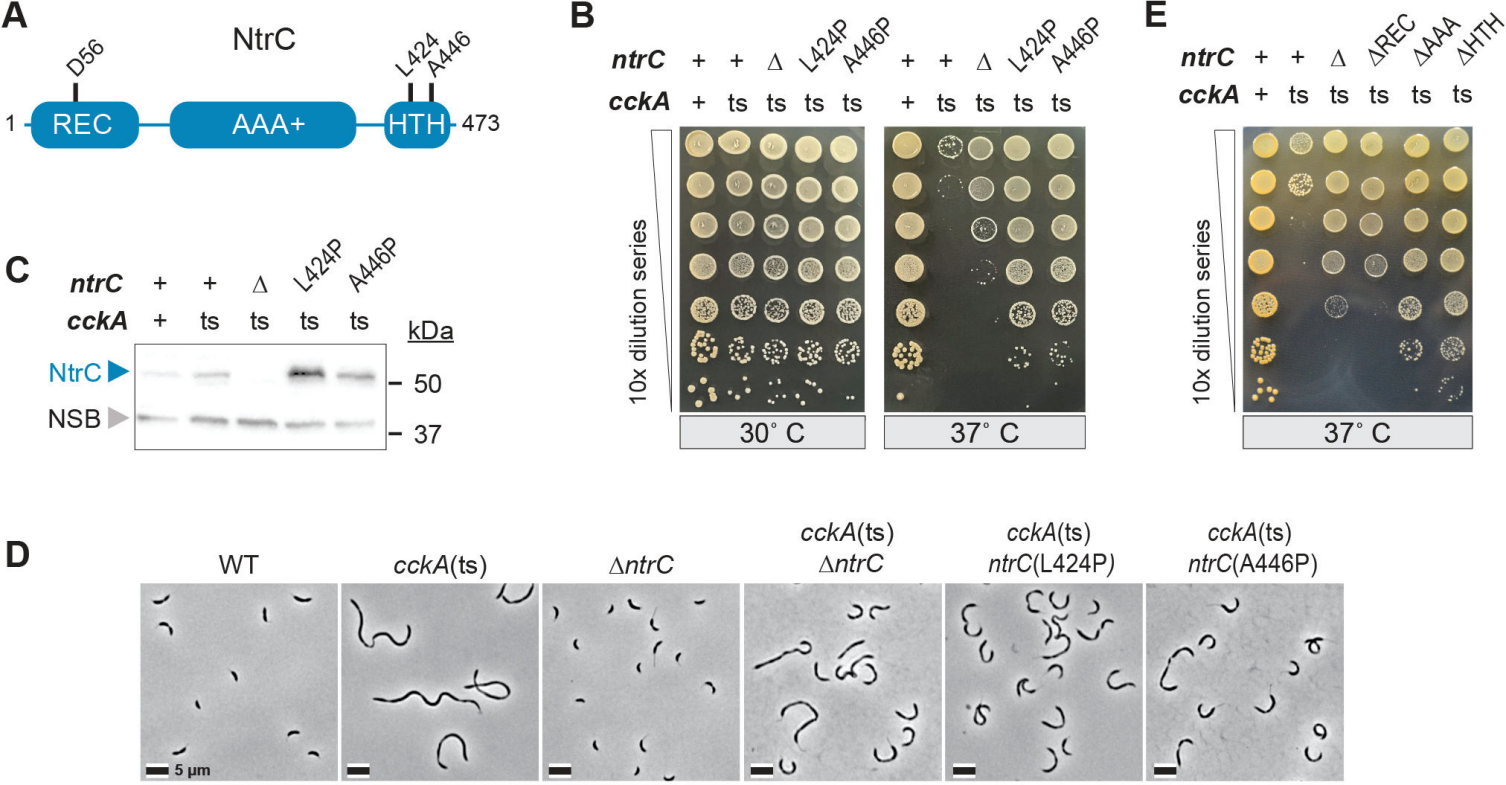
A tiered pattern of genetic suppression in *Caulobacter*, where loss of CckA function is variably rescued by structurally distinct *ntrC* loss-of-function mutations. (**A**) Model of *Caulobacter* NtrC protein showing the receiver (REC) domain and the site of aspartyl phosphorylation on residue D56, the AAA+ ATPase domain, and the DNA-binding helix-turn-helix (HTH) domain. (**B**) Serial dilutions of *Caulobacter* strains encoding wild-type (+) or mutant alleles of *cckA* and *ntrC* grown at the indicated temperatures. Temperature-sensitive mutant of *cckA*(ts), in-frame deletion of *ntrC* (∆), and *ntrC* HTH domain point mutants (L424P or A446P) are indicated. (**C**) Western blot of lysates prepared from wild-type (+) and mutant strains shown in panel B, probed with polyclonal α-NtrC antiserum. A non-specific band (NSB) is indicated. Strains were grown at the restrictive temperature (37°C) for 24 h prior to lysate preparation. (**D**) Phase contrast light micrographs of WT and mutant strains of *Caulobacter* grown at the restrictive temperature for 3.25 h. Scale bar is 5 µm. Cell length quantification is shown in Fig. S1. (**E**) Serial dilutions of *Caulobacter* strains encoding wild-type (+) or mutant alleles of *cckA* (as above) and *ntrC* (in-frame deletion [∆] or lacking individual HTH, AAA+, or REC domains). See Fig. S4 for paired control titers grown at the permissive temperature (30°C).

Shifting a culture of a temperature-sensitive *Caulobacter cckA* mutant [*cckA*(ts)] (19) from a growth-permissive temperature (30°C) to a growth-restrictive temperature (37°C) resulted in expected phenotypes, including cell filamentation and loss of colony formation on complex (peptone yeast extract [PYE]) solid medium (19) (Fig. 1C and 2D; Fig. S1). However, rare colonies grew at the restrictive temperature. We picked several of these apparent suppressor mutants and confirmed that they grew at the restrictive temperature despite harboring the genetic lesions in the *cckA* ATPase domain region (*cckA*[I484N, P485A]) that cause temperature sensitivity (19). Through whole-genome sequencing, we identified mutations that putatively suppress the *cckA*(ts) lesions, including a second-site mutation in the *cckA* HisKA dimerization/phosphoacceptor domain (R334S) (Fig. 1B; Table S1).

Several extragenic mutations in genes associated with the CckA cell cycle signaling pathway were identified through this selection, including multiple strains with mutations in *ctrA* (Fig. 1B; Table S1), the essential RR (39) and phosphoryl-transfer target of CckA (19). Among these were four independent isolates harboring a *ctrA*(D8G) mutation and one harboring *ctrA*(T90A). The *ctrA*(D8G) mutation has been previously characterized as a gain-of-function allele that allows *Caulobacter* to grow in the absence of *cckA* (33). Additionally, we identified putative loss-of-function mutations in *cpdR*, which is also a phosphorylation target of CckA and regulates the proteolytic stability of CtrA (23, 40) (Fig. 1B; Table S1). Loss of CpdR function disrupts its role in targeting CtrA for degradation, thereby enhancing CtrA stability in the cell (23). To validate *cpdR* as a suppressing target of *cckA*(ts) temperature sensitivity, we constructed a *cpdR* in-frame deletion mutation (∆*cpdR*) in a *cckA*(ts) genetic background. Deletion of *cpdR* fully suppressed the temperature sensitivity of *cckA*(ts), restoring growth/colony forming units (CFUs) at the restrictive temperature (37°C) to a level comparable to that observed for the group of *cpdR* point mutant alleles isolated in our genetic selection (Fig. 1C). These results reveal a pattern of suppression whereby loss of CckA function is mitigated by mutations that enhance CtrA activity or its proteolytic stability.

### Suppressing mutations outside of the known CckA regulatory axis

In addition to mutations that are on the pathway, we identified mutations in genes that are not known to be a part of the established *Caulobacter* cell cycle regulatory network, pointing to possible alternative mechanisms that can mitigate loss of CckA function. For example, multiple independent mutations in the beta subunit of RNA polymerase (*rpoB*) were associated with rescued colony growth of *cckA*(ts) strains on dilution plates at the restrictive temperature (Fig. 1B; Table S1). A prior study demonstrated that a *rpoB* mutation enhances CtrA promoter occupancy, likely via increased (p)ppGpp signaling (41). The identification of *rpoB* mutations as suppressors of *cckA*(ts) suggests that altered RNA polymerase activity may modulate CtrA function, or the transcription of CtrA targets, independent of direct phosphorelay input. Mutations in *CCNA_01689*, which encodes inosine-5′-monophosphate dehydrogenase (an enzyme that catalyzes the rate-limiting step in guanine nucleotide synthesis), were also linked to the rescue of the *cckA*(ts) phenotype. In addition, mutations in the LysR-family transcriptional regulator *CCNA_02227* and the cell envelope regulator *cenR* were similarly associated with *cckA*(ts) suppression (Fig. 1B; Table S1). Finally, we identified three independent suppressor isolates carrying mutations in the DNA-binding domain of the bEBP gene, *ntrC* (Fig. 1B; Table S1). We conclude that a diverse set of mutations can suppress the *cckA*(ts) phenotype, including second-site modifications of *cckA* itself, mutations within the signaling pathway it regulates, and mutations in genes with broader roles in transcription and nucleotide synthesis.

### Loss-of-function mutations in the DNA-binding HTH domain of *ntrC* fully suppress *cckA*(ts) temperature sensitivity

Our genetic selection uncovered two distinct point mutations in *ntrC* that were associated with increased CFUs of a *cckA*(ts) mutant on dilution plates at the restrictive temperature (Fig. 1B). These mutations, A446P and L424P, are located within the DNA-binding helix-turn-helix (HTH) domain of *ntrC* (Fig. 2A). Since a role for *ntrC* in the CckA-ChpT-CtrA phosphorelay had not been previously described, we prioritized *ntrC* for further investigation.

To directly demonstrate that the A446P and L424P alleles of *ntrC* suppress the *cckA*(ts) phenotype, we replaced wild-type *ntrC* with either *ntrC*(L424P) or *ntrC*(A446P) in a *cckA*(ts) genetic background. Both mutant *ntrC* alleles restored the growth/viability of the *cckA*(ts) mutant to wild-type (WT) levels on complex solid medium at the restrictive temperature (37°C) without impacting viability at the permissive temperature (30°C) (Fig. 2B). These *ntrC* alleles partially rescued the filamentation and cell division defects of the *cckA*(ts) mutant at the restrictive temperature (Fig. 2D; Fig. S1). We hypothesized that proline substitutions at residues 424 and 446 disrupt NtrC function because these mutations occur within modeled α-helices 1 and 2 of the DNA-binding HTH domain (Fig. S2) (42). Proline has the highest helix-breaking propensity of the 20 standard amino acids (43, 44), and the structural integrity of HTH helices is essential for DNA binding, a property central to NtrC function (37).

Loss of *Caulobacter ntrC* function results in several distinct phenotypes, including an inability to grow in defined medium (M2G) with ammonium (NH_4_^+^) as the sole nitrogen source, hyper-elongated polar stalks, and mucoidy on complex (PYE) medium. These phenotypes are chemically complemented by adding the nitrogen source glutamine to the medium (37). We introduced the *ntrC*(A446P) and *ntrC*(L424P) alleles into an otherwise wild-type *Caulobacter* background via allele replacement, and these mutant strains phenocopied a ∆*ntrC* strain. Specifically, both of these HTH domain point mutants failed to grow with NH_4_^+^ as the sole nitrogen source, and growth was restored when NH_4_^+^ was replaced with a molar-equivalent concentration of glutamine (Fig. S3). Additionally, both mutants exhibited elongated stalks and a mucoid cell pellet, similar to the *ntrC* deletion strain (Fig. S3). Both mutant proteins were stably expressed in a *cckA*(ts) background (Fig. 2C), indicating that these loss-of-function phenotypes are not due to reduced protein expression or instability. In fact, mutant NtrC proteins were expressed at higher levels than wild-type NtrC, consistent with our previous report that *Caulobacter* NtrC is an autorepressor (37). We conclude that the A446P and L424P mutations in the DNA-binding HTH domain of NtrC result in a loss of protein function.

### Distinct contributions of the NtrC domains to *cckA*(ts) suppression: a key role for the REC domain

Given the loss-of-function phenotypes observed in *ntrC*(L424P) and *ntrC*(A446P) mutants (Fig. S3), we predicted that complete deletion of *ntrC* would similarly suppress the temperature sensitivity of *cckA*(ts) at the restrictive temperature. Contrary to our expectation, *ntrC* deletion (∆*ntrC*) only partially rescued the growth defect of *cckA*(ts) as shown by serial dilution assays at the restrictive temperature (Fig. 2B). The *∆ntrC* allele alleviated the filamentation and cell division defects of *cckA*(ts) to a similar extent as the *ntrC*(L424P) and *ntrC*(A446P) point mutants (Fig. 2D; Fig. S1). Since these point mutations reside in the DNA-binding HTH domain of NtrC (Fig. 2A), we hypothesized that deleting the entire HTH domain would replicate their suppressive effects. Consistent with this prediction, deletion of the *ntrC* HTH domain [*ntrC*(∆HTH)] nearly fully restored growth of *cckA*(ts) at the restrictive temperature (Fig. 2E; Fig. S4). These results indicate that disruption of NtrC DNA binding is sufficient to fully suppress the loss of CckA function.

NtrC, a bEBP (45, 46), consists of three functional domains: (i) a two-component receiver (REC) domain, (ii) an ATPase associated with cellular activity (AAA+) domain, and (iii) a DNA-binding HTH domain (Fig. 2A). To assess the specific contributions of the REC and AAA+ domains to *cckA*(ts) suppression, we introduced domain deletions in *ntrC* within the *cckA*(ts) background. The *ntrC*(∆AAA) allele improved *cckA*(ts) growth but not as effectively as *ntrC*(∆HTH). In contrast, deleting the REC domain [*ntrC*(∆REC)] only partially suppressed growth defects, similar to ∆*ntrC* (Fig. 2E). Taken together, these data indicate that suppression is most robust when NtrC retains an intact REC and AAA+ domain, as seen in the *ntrC*(L424P), *ntrC*(A446P), and *ntrC* (∆HTH) mutants. Notably, we have previously shown that these NtrC domain mutants (∆HTH, ∆REC, and ∆AAA) are stably expressed in *Caulobacter* at significantly higher levels than wild-type NtrC (37). The possible impact of increased steady-state levels of these mutant alleles on gene expression is discussed in sections below. These results illuminate a critical role for the NtrC REC domain in the *cckA*(ts) suppression mechanism.

**Fig 3:**
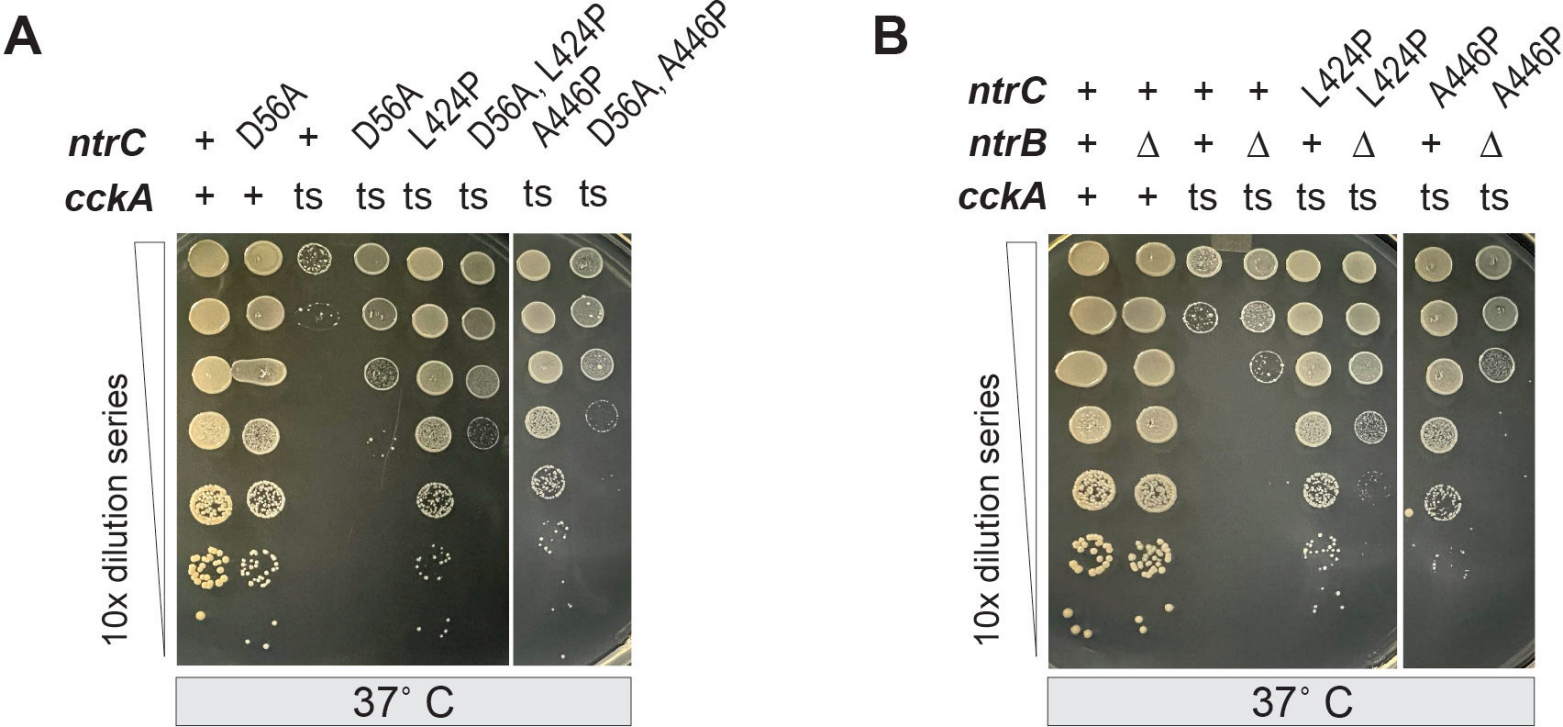
Phosphorylation of the NtrC receiver domain by NtrB is required for *cckA*(ts) growth rescue. Serial dilutions of *Caulobacter* strains encoding wild-type (+) or mutant alleles of *cckA*, *ntrC,* and/or *ntrB* cultivated at the restrictive (37°C) temperature. (**A**) Temperature-sensitive *cckA* allele and *ntrC* point mutants (D56A, L424P, A446P; single and in combination) are marked. (**B**) Temperature-sensitive *cckA* allele, in-frame deletion of *ntrB* (∆), and *ntrC* point mutants (L424P or A446P) are marked. See Fig. S4 for paired control titers grown at the permissive temperature (30°C).

Phosphorylation of the NtrC REC domain triggers conformational changes that regulate its activity (47). Previously, we demonstrated that phosphorylation of the conserved aspartate residue (D56) in the REC domain is required for *Caulobacter* growth on NH₄^+^ as the sole nitrogen source (37). To test whether REC phosphorylation is necessary for *cckA*(ts) suppression, we introduced the non-phosphorylatable *ntrC*(D56A) allele into the *cckA*(ts) background. Similar to *ntrC*(∆REC), the *ntrC*(D56A) mutation only partially suppressed *cckA*(ts) temperature sensitivity (Fig. 3A). We further tested whether phosphorylation was required for suppression by *ntrC* HTH domain mutants by generating *ntrC*(D56A, L424P) and *ntrC*(D56A, A446P) double mutants. Suppression in these strains was comparable to *ntrC*(D56A), indicating that phosphorylation of the REC domain is required for full suppression by *ntrC*(L424P) and *ntrC*(A446P) (Fig. 3A; Fig. S4).

Given this result, we predicted that deleting *ntrB*(∆*ntrB*), which encodes the cognate kinase of NtrC (37), would similarly impair suppression of *cckA*(ts) by *ntrC*(L424P) and *ntrC*(A446P). As expected, *∆ntrB* attenuated suppression by these *ntrC* mutant alleles, further supporting a critical role for NtrC phosphorylation in this mechanism (Fig. 3B; Fig. S4). Together, these results show that the function of CckA can be fully bypassed through an NtrC mutant that cannot bind DNA, but that has an intact and phosphorylatable REC domain.

### Glutamine abrogates *cckA*(ts) suppression by *ntrC* mutations

**Fig 4:**
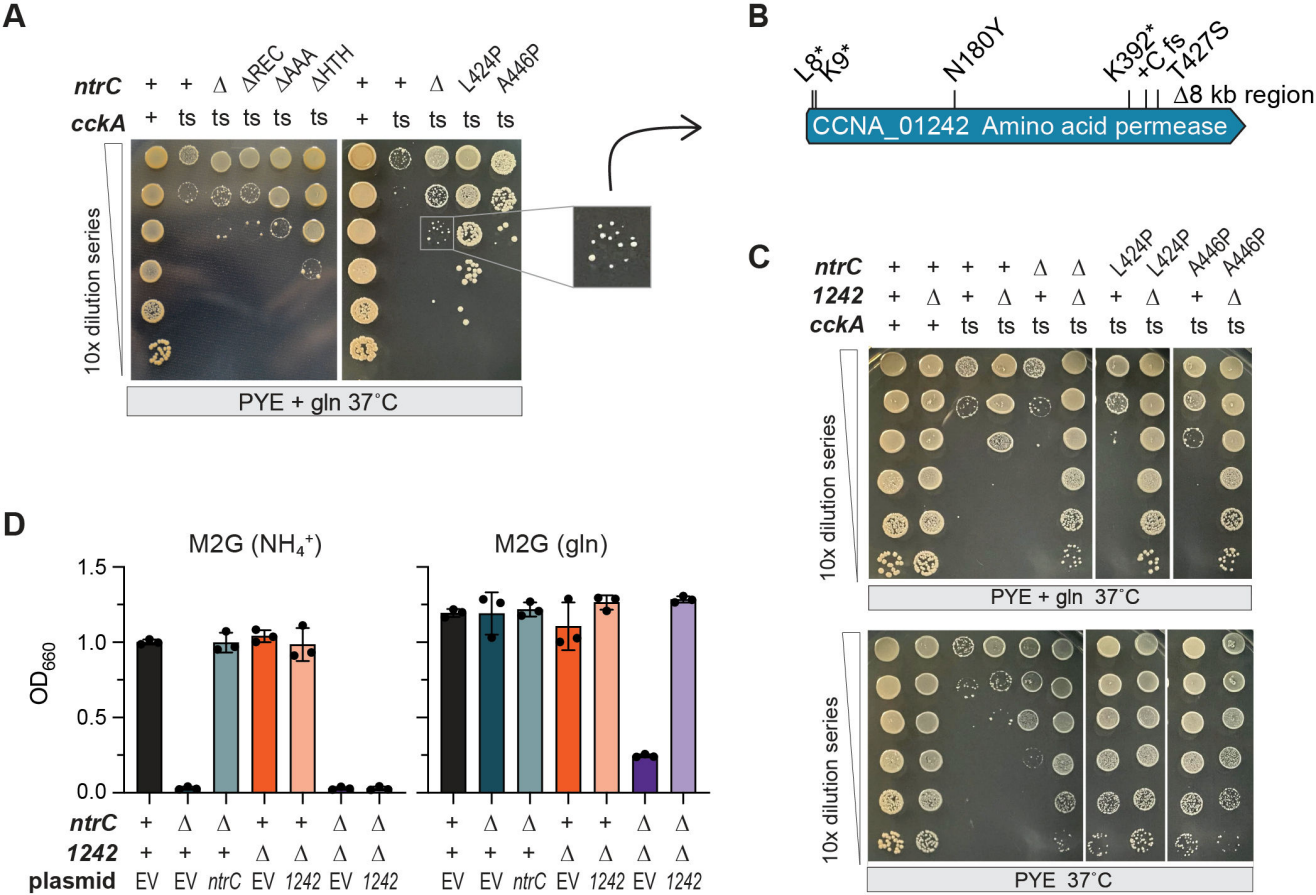
Glutamine abrogates ntrC-mediated suppression of cckA(ts), a role for CCNA_01242 in glutamine transport. (**A**) Temperature-sensitive mutant of *cckA*(ts), in-frame deletion of *ntrC*(∆), *ntrC* mutants lacking an HTH, AAA+, or REC domains, and *ntrC* point mutants (L424P and A446P) (as indicated) were titered on agar supplemented with 9.3 mM glutamine (gln). See Fig. S4 for paired control titers grown at the permissive temperature (30°C). The colony inset illustrates the selection approach used to isolate “glutamine-blind” mutants, in which *ntrC*-mediated suppression of *cckA*(ts) was restored despite the presence of glutamine. (**B**) Whole-genome sequencing of 10 such mutants identified deletion, frameshift, nonsense, and point mutations in locus *CCNA_01242*, a gene encoding a predicted amino acid permease. (**C**) Log_10_ serial dilutions of *Caulobacter* strains encoding wild-type (+) or mutant alleles of *cckA*, *ntrC,* and *CCNA_01242*. Temperature-sensitive mutant of *cckA*(ts), in-frame deletion of *CCNA_01242* or *ntrC* (∆), and *ntrC* point mutants (L424P and A446P) are marked. Strains were cultivated at the restrictive temperature (37°C) in the presence and absence of 9.3 mM glutamine (gln). See Fig. S4 for paired control titers grown at the permissive temperature (30°C). (**D**) Culture density of *ntrC* and *CCNA_01242* deletion mutants (∆) after 24 h of growth in defined M2-glucose (M2G) medium with either ammonium (NH_4_^+^) or glutamine (gln) as the sole nitrogen source. Genetic complementation of *ntrC* or *CCNA_01242* from a plasmid is indicated. EV, empty vector control plasmid.

NtrC activates transcription of glutamine synthetase (*glnA*) (37), which is predicted to provide the sole route of inorganic nitrogen assimilation in *Caulobacter* (48, 49). Mutations in the NtrC HTH domain (L424P or A446P), as well as deletions of the HTH, REC, and AAA domains, disrupt NtrC function and suppress the temperature sensitivity of *cckA*(ts) to varying extents (Fig. 2). Given that supplementation with glutamine alleviates the phenotypes associated with loss of *ntrC* function and broadly restores transcriptional balance in a ∆*ntrC* mutant (37), we hypothesized that suppression of *cckA*(ts) by *ntrC* mutations is at least partially mediated by reduced intracellular glutamine levels. Accordingly, we predicted that restoring cellular glutamine through exogenous supplementation would negate the suppressive effects of *ntrC* mutations. Consistent with this prediction, supplementation with 9.3 mM glutamine had no impact on strain viability at 30°C but significantly impaired the ability of all *ntrC* mutations to rescue the growth defect of *cckA*(ts) at 37°C (Fig. 4A; Fig. S4). We conclude that intracellular glutamine limitation contributes to *ntrC*-mediated suppression of the *cckA*(ts) phenotype.

To further investigate the role of glutamine in *ntrC*-mediated suppression of *cckA*(ts), we isolated spontaneous mutant strains that restored the ability of mutant *ntrC* alleles to suppress the temperature sensitivity of *cckA*(ts) in media containing glutamine (Fig. 4A and B). We sequenced genomes of 10 of these “glutamine-blind” mutants; eight harbored frameshift or point mutations in locus *CCNA_01242*, a gene encoding an annotated amino acid permease, and a ninth harbored an 8-kb deletion that included *CCNA_01242* (Fig. 4B; Table S2). The nature of the mutations in this gene (e.g., nonsense, frameshift, and deletion) strongly suggested they resulted in a loss of function. Supporting this model, an in-frame deletion of *CCNA_01242* (∆*CCNA_01242*) restored growth at the restrictive temperature of *cckA*(ts) strains carrying suppressing *ntrC* mutant alleles (∆, L424P, or A446P) in the presence of glutamine (Fig. 4C). Deletion of *CCNA_01242* enhanced growth and viability of *cckA*(ts) strains on dilution plates by approximately one log_10_ unit. While ∆*ntrC* alone only partially rescued the temperature-sensitive phenotype of *cckA*(ts) grown on PYE (without glutamine supplementation), combining ∆*CCNA_01242* with ∆*ntrC* conferred full rescue of *cckA*(ts). Notably, loss of *CCNA_01242* has previously been associated with increased fitness across a range of complex and defined media conditions (50, 51), though the mechanisms remain undefined.

As deletion of *CCNA_01242* rendered suppressed strains insensitive to extracellular glutamine, we hypothesized that this gene encodes a protein that is competent to transport glutamine at a concentration of 9.3 mM. To test this, we cultivated a *CCNA_01242* in-frame deletion strain in defined medium with either NH_4_^+^ or glutamine as the sole nitrogen source. As previously demonstrated (37), the ∆*ntrC* strain grew when glutamine was the sole nitrogen source but failed to grow with NH_4_^+^ (Fig. 4D). However, the ability of this mutant to utilize glutamine depended on the amino acid permease encoded by *CCNA_01242*, as the ∆*ntrC* ∆*CCNA_01242* double mutant was unable to grow in glutamine-containing medium (Fig. 4D). These results provide evidence that CCNA_01242 can transport glutamine and support a model in which glutamine uptake through this permease prevents loss-of-function *ntrC* mutations from suppressing the *cckA*(ts) phenotype when extracellular glutamine concentration is high (9.3 mM).

### Loss of *ntrC* function elevates (p)ppGpp and sustains CtrA protein levels to support *cckA*(ts) suppression

**Fig 5:**
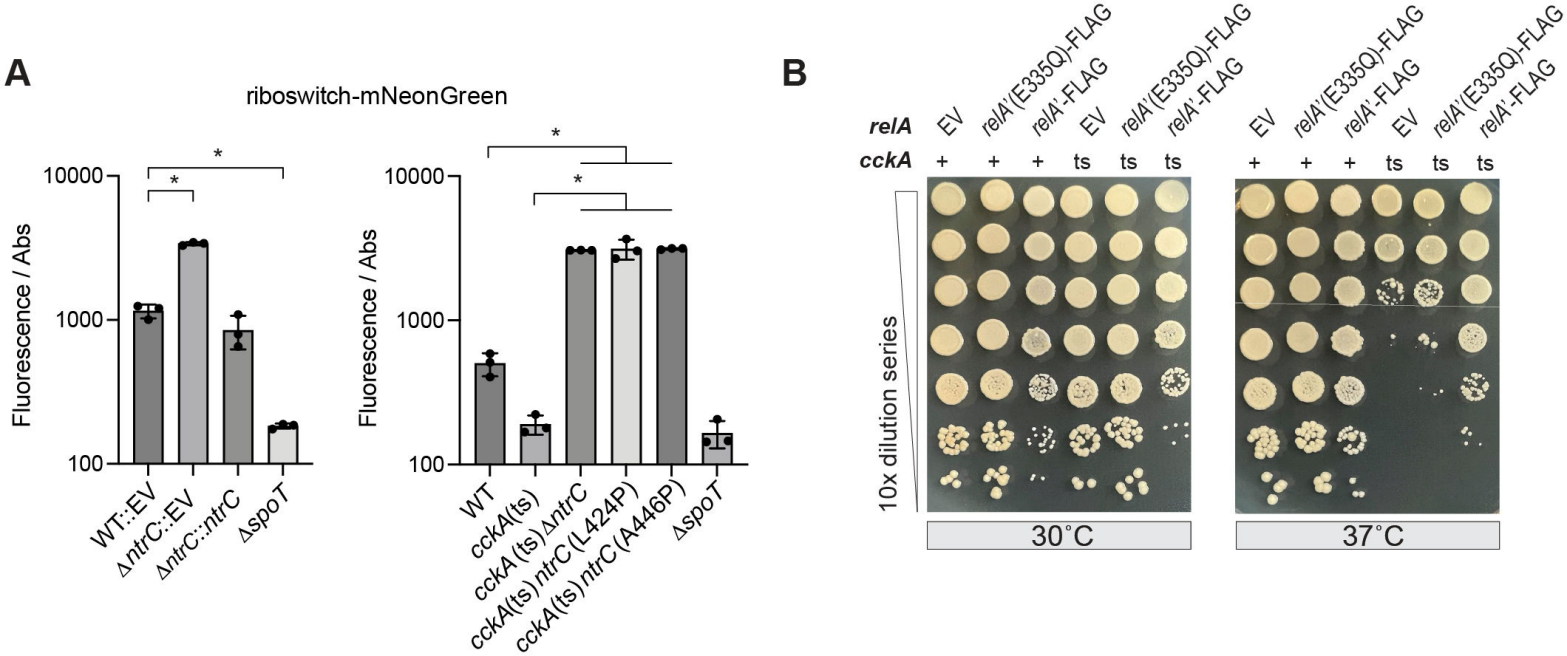
Elevated ppGpp levels are associated with suppression of the *cckA*(ts) phenotype. (**A**) ppGpp levels in *ntrC* mutant backgrounds were measured by fusing the *D. hafniense ilvE* ppGpp-sensing riboswitch (54) to *mNeonGreen*. Fluorescence signal was normalized to cell density as measured by absorbance at 660 nm (Abs). See Materials and Methods for riboswitch induction and cell cultivation conditions. Left: WT and ∆*ntrC* strains carrying an empty complementation vector (EV), the genetically complemented *∆ntrC::ntrC* strain, and a strain lacking the sole (p)ppGpp synthetase in *Caulobacter* (∆*spoT)*. Right: WT, *cckA*(ts), *cckA*(ts) harboring labeled mutations in *ntrC*, and the ∆*spoT* negative control strain. Data represent mean ± standard deviation of three replicates. Statistical significance was determined by one-way ANOVA followed by Tukey’s multiple comparison test (*P 0.0001). Significant comparisons to WT or *cckA*(ts) strains are shown. (**B**) Serial dilutions of *Caulobacter* strains harboring wild-type (+) or mutant (ts) *cckA* and expressing a constitutively active synthetase version of *Escherichia coli relA* (*relA*′-FLAG), a catalytically inactive mutant [*relA*′(E335Q)*-*FLAG], or EV grown at the permissive (30°C) and restrictive (37°C) temperatures. Agar was supplemented with 0.3% xylose (xyl) to induce *relA* expression.

** **In *Caulobacter*, elevated intracellular glutamine suppresses the synthesis of the alarmone nucleotide (p)ppGpp (48), a global regulator of cell physiology (52, 53). Given this relationship, and the knowledge that *ntrC* mutants cannot assimilate NH₄^+^ into glutamine (37), we hypothesized that loss of *ntrC* function leads to increased (p)ppGpp levels. To test this, we fused a ppGpp-regulated riboswitch from *Desulfitobacterium hafniense* (54) to *mNeonGreen* (55) following a strategy similar to the RNA aptamer-based (p)ppGpp reporter developed by Sun et al. (56). This riboswitch was recently shown to reliably report intracellular (p)ppGpp levels when fused to an unstable luminescent reporter in *Bacillus subtilis* (57). Based on this result, we reasoned that (p)ppGpp in *Caulobacter* would promote transcriptional readthrough of the riboswitch into *mNeonGreen*, providing a fluorescent readout that reflected intracellular (p)ppGpp levels. Indeed, fluorescence was nearly undetectable in a *spoT* deletion strain, which lacks the sole (p)ppGpp synthetase in *Caulobacter* (58) (Fig. 5A). In contrast, fluorescence signal was significantly higher in a ∆*ntrC* strain compared to WT, and this signal was restored to wild-type levels when a complementing copy of *ntrC* was integrated at an ectopic locus (Fig. 5A). Similarly, *cckA*(ts) strains carrying *ntrC* loss-of-function point mutations (L424P or A446P) exhibited fluorescence levels comparable to the ∆*ntrC* mutant (Fig. 5A). These results support a model in which loss of *ntrC* function leads to elevated intracellular (p)ppGpp levels.

 Elevated (p)ppGpp levels have been reported to sustain CtrA protein levels after transcriptional shut-off through an unknown post-transcriptional mechanism (59). Given that *cpdR* loss-of-function mutations, which stabilize CtrA protein (23), rescue the temperature sensitivity of *cckA*(ts) (Fig. 1C), we hypothesized that elevated (p)ppGpp levels similarly suppress *cckA*(ts) in *ntrC* mutants by promoting CtrA expression or stability. To begin testing this idea, we first assessed whether (p)ppGpp *per se* contributed to *cckA*(ts) suppression by expressing an *Escherichia coli* RelA variant (*relA*′-FLAG) that lacks hydrolase activity and constitutively synthesizes (p)ppGpp (60). This allele is known to enhance (p)ppGpp levels and increase CtrA abundance in *Caulobacter* (59). As expected, *relA*′-FLAG expression reduced colony size at the permissive temperature (30°C) in both WT and *cckA*(ts) strains, consistent with the growth-inhibitory effects of high (p)ppGpp (Fig. 5B). At the restrictive temperature (37°C), *relA*′-FLAG expression enhanced *cckA*(ts) CFUs by approximately two log_10_ units, supporting our model. In contrast, the expression of a catalytically inactive mutant [*relA*′(E335Q)-FLAG] (59) failed to enhance CFUs at the restrictive temperature (Fig. 5B), which directly implicated (p)ppGpp in the suppression mechanism. These data support a model in which elevated (p)ppGpp synthesis upon loss of *ntrC* function enables bypass of CckA function.

**Fig 6:**
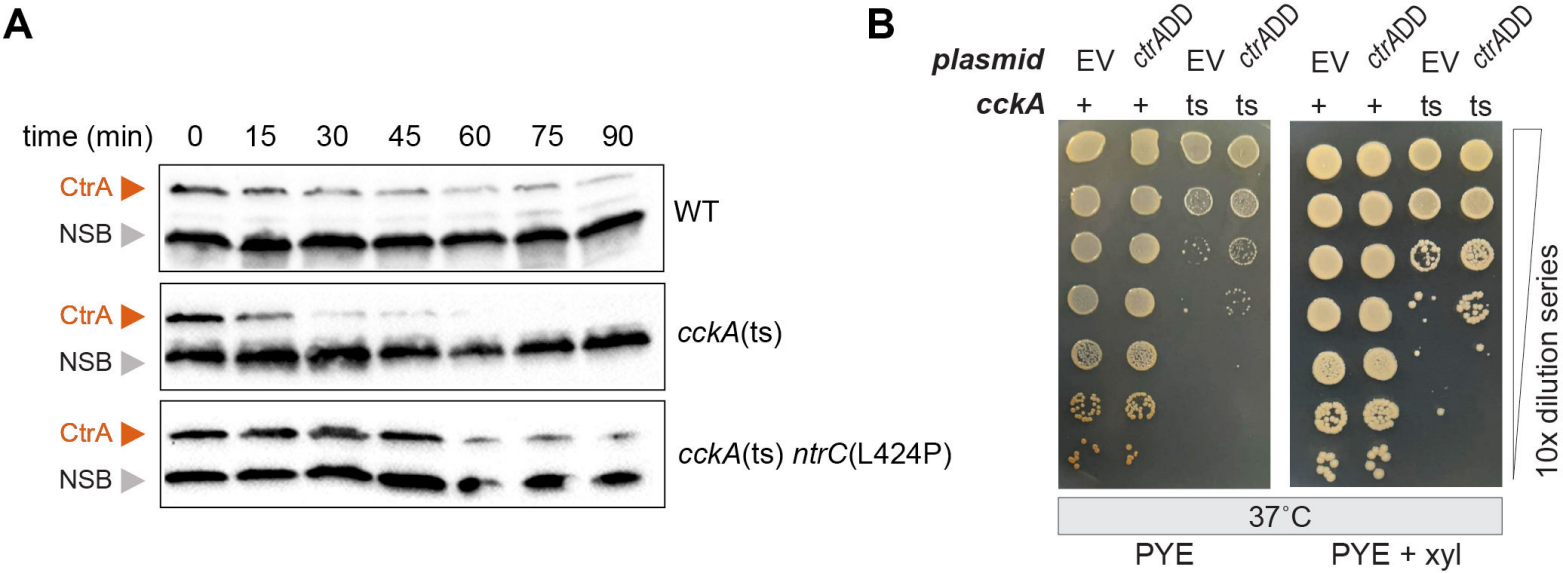
Stabilization of CtrA protein as a route to suppress temperature sensitivity of *cckA*(ts). (**A**) CtrA levels in WT, *cckA*(ts), and *cckA*(ts) *ntrC*(L424P) monitored by immunoblot after the addition of rifampin (time 0) to shut off CtrA expression. Cells were shifted to the restrictive temperature (37°C) for 3.25 h before rifampin treatment. NSB, non-specific band that reacts with polyclonal serum. (**B**) Serial dilution of *Caulobacter* strains encoding wild-type (+) or mutant (ts) alleles of *cckA* that harbor either empty vector (EV) or the *ctrA*DD (39, 62) overexpression construct. Dilution series were spotted onto PYE and PYE supplemented with inducer (0.3% final concentration xylose [xyl]) and grown at the restrictive temperature (37°C).

To further test this model, we assessed CtrA protein levels in WT, *cckA*(ts), and *cckA*(ts) *ntrC*(L424P) strains at the restrictive temperature after CtrA expression shutoff. Specifically, we inhibited *ctrA* transcription with rifampicin (59, 61) and monitored CtrA protein levels over time. As previously reported (18, 23), CtrA levels declined more rapidly in *cckA*(ts) than in WT. In contrast, CtrA levels in the *cckA*(ts) *ntrC*(L424P) strain were maintained at near-WT levels following transcriptional shutoff (Fig. 6A; Fig. S5). These results indicate that suppression of *cckA*(ts) by *ntrC* mutation is associated with sustained CtrA levels in the cell, either through enhanced *ctrA* mRNA translation or reduced CtrA degradation. This mechanism aligns with the observation that *cpdR* loss-of-function mutations, which block regulated CtrA proteolysis (23, 61), can similarly bypass the essential kinase activity of CckA (Fig. 1).

 We then sought to test whether direct stabilization of CtrA protein would similarly suppress the *cckA*(ts) phenotype. To address this question, we expressed *ctrA*DD, a previously characterized allele in which the C-terminal Ala-Ala degradation sequence is mutated to Asp-Asp, resulting in a stabilized form of CtrA (39, 62). As predicted, *ctrA*DD expression enhanced *cckA*(ts) CFUs by approximately one log_10_ unit at 37°C (Fig. 6B). However, the degree of suppression was weaker than observed with *relA*′-FLAG (Fig. 5B) or ∆*cpdR* (Fig. 1C), suggesting that stabilization of CtrA protein through mutation of its C-terminal degradation sequence is not sufficient to fully bypass the requirement for CckA function.

### Beyond *ctrA: gdhZ* contributes to *cckA*(ts) suppression by *cpdR* deletion

Since stabilization of CtrA protein alone did not fully suppress the *cckA*(ts) phenotype, we tested whether other cell cycle regulators (whose degradation is promoted by CpdR) contribute to the strong *cckA*(ts) suppression observed upon *cpdR* deletion (Fig. 1C). We focused on KidO (63) and GdhZ (64), which function together at a similar stage of the cell cycle to influence cell division (64) and are known to be stabilized upon *cpdR* deletion. Although both proteins have roles in cell cycle regulation, their biochemical activities are distinct: GdhZ catalyzes the conversion of glutamate to α-ketoglutarate and therefore directly impacts nitrogen and central carbon metabolism; KidO binds NAD(H) but has no known link to core metabolic pathways. To test whether stabilization of these proteins contributes to the rescue of *cckA*(ts) upon deletion of *cpdR*, we deleted *kidO* or *gdhZ* in the *cckA*(ts)∆*cpdR* background. Deletion of *kidO* had no effect on the suppression of the *cckA*(ts) phenotype, while deletion of *gdhZ* blunted the suppressive effect of *cpdR* deletion (Fig. S6). Given the role of GdhZ in converting glutamate to α-ketoglutarate, its accumulation in the ∆*cpdR* background is predicted to enhance glutamate conversion to α-ketoglutarate, possibly limiting glutamine biosynthesis. These results are consistent with a model in which changes in glutamine, glutamate, and α-ketoglutarate levels (or flux) contribute to *cckA*(ts) suppression in both *cpdR* and *ntrC* mutant contexts, though additional studies are needed to validate this model.

### A transcriptome-level view of a synthetic rescue interaction

**Fig 7:**
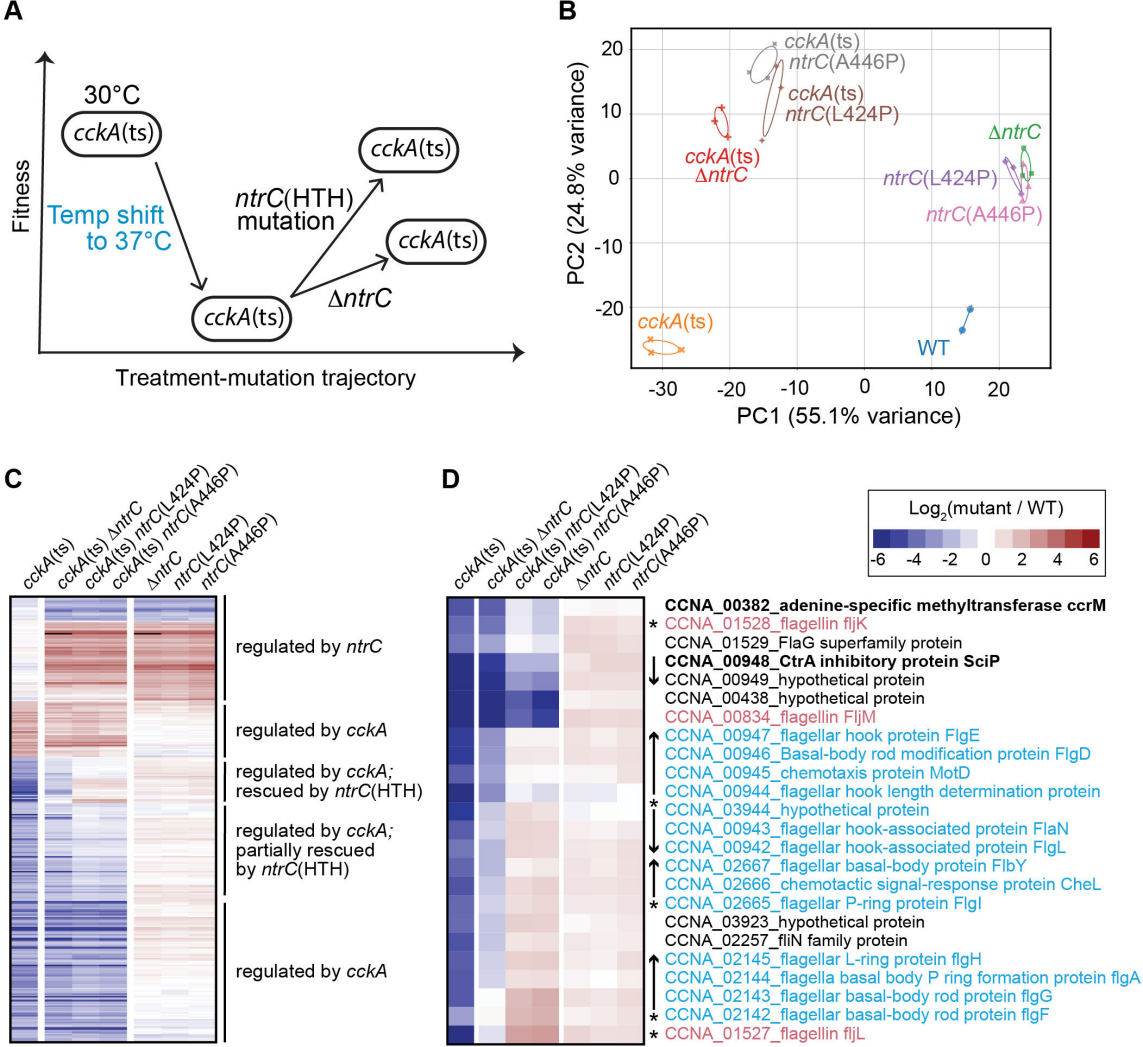
Transcriptomic analysis of a synthetic rescue interaction between *ntrC* and the essential sensor kinase *cckA*. (**A**) Schema illustrating the fitness consequences of shifting the *cckA*(ts) strain to the restrictive temperature (37°C) and the variable suppression of temperature sensitivity by *ntrC* mutations. The temperature shift reduces fitness of *cckA*(ts), and complete *ntrC* deletion (∆*ntrC*) partially restores fitness. Mutations in the DNA-binding helix-turn-helix domain of *ntrC* (∆HTH, L424P, and A446P) more robustly rescue fitness of *cckA*(ts) at the restrictive temperature. (**B**) Principal component analysis of transcriptomic data sets of strains harboring *ntrC* mutations (∆*ntrC*, L424P, and A446P) in either a WT or a *cckA*(ts) mutant background. The first two principal axes (PC1 and PC2) are shown. (**C**) Hierarchically clustered heatmap displays the 324 genes differentially expressed in either the *cckA*(ts) or ∆*ntrC* backgrounds (see Table S3 for genes in this cluster), highlighting transcriptional differences between *cckA*(ts) and the *cckA*(ts) *ntrC* mutant (i.e., rescued) strains. Genes included in the heatmap met the criteria of ∣Fold Change∣ > 3 and an FDR-adjusted *P*-value < 10^−6^ in either *cckA*(ts) or ∆*ntrC* compared to WT. (**D**) Heatmap highlighting genes with the largest transcriptional differences between *cckA*(ts) strains harboring the *ntrC* HTH point mutations (L424P and A446P) and the *cckA*(ts) strain with a ∆*ntrC* deletion. Class III flagellar genes are marked in cyan, and Class IV flagellar genes are marked in salmon. Genes were hierarchically clustered and then manually arranged to reflect operon arrangements. Arrows indicate operon structures, and asterisks denote genes with FlbD binding sites in their promoter as identified by Fumeaux et al. (33). Heatmap colors represent the log_2_(mutant/WT) expression for the mutant strain indicated above the heatmap. Expression values for the genes highlighted in panels C and D can also be found in Table S3.

The results reported so far reveal a tiered pattern of suppression in *Caulobacter*, whereby temperature-sensitive loss-of-function mutations in the essential cell cycle regulatory kinase CckA are variably rescued by structurally distinct loss-of-function mutations in *ntrC* (Fig. 7A). Given that NtrC is a bEBP, we hypothesized several mechanisms through which *ntrC* mutations might rescue *cckA*(ts) conditional lethality by altering transcription: (i) *ntrC* mutations broadly restore gene expression in the *cckA*(ts) background to wild-type levels, (ii) *ntrC* mutations result in a distinct gene expression profile unrelated to either WT or the primary *cckA*(ts) mutant, or (iii) a combination of these effects. To test these models, we performed RNA sequencing (RNA-seq) to evaluate the transcriptional impact of *cckA*(ts) and *ntrC* mutations, both individually and in combination (Table S3).

Principal component analysis (PCA) of the transcriptomic data revealed that PC1 and PC2 together accounted for 80% of the total variance in transcription (Fig. 7B). These principal axes can be ascribed to transcriptional dysregulation caused by loss of *cckA* function (PC1) and *ntrC* function (PC2). Consistent with their growth, stalk length, and mucoidy phenotypes (Fig. S3), the transcriptional profiles of the *ntrC*(L424P) and *ntrC*(A446P) mutants clustered closely with ∆*ntrC* on the ordination plot (Fig. 7B). When combined, the transcriptional effects of *cckA*(ts) and the *ntrC* mutations were largely independent, given the clustering of the double mutant transcriptomes near the diagonal of PC1 and PC2. However, compared to the parental *cckA*(ts) strain, the double mutants are modestly shifted toward WT on PC1. The *ntrC* HTH domain point mutants (L424P and A446P) shifted the *cckA*(ts) transcriptome even closer to WT on PC1 than deletion of *ntrC* (Fig. 7B). This trend aligns with the stronger rescue phenotypes observed for these point mutants compared to ∆*ntrC* (Fig. 2B and 7A), suggesting their more robust restoration of gene expression patterns disrupted by *cckA*(ts).

Using a conservative statistical cutoff, we identified 247 CckA-regulated genes and 78 NtrC-regulated genes (Table S3). A combined and clustered data set containing both CckA and NtrC regulons revealed only one overlapping gene, resulting in a set of 324 genes that are dysregulated by mutation of *cckA* or *ntrC* (Fig. 7C; Table S3). Genes with significantly decreased transcription upon loss of *cckA* function are consistent with published *cckA*(ts) transcriptomic data (18) and include numerous cell cycle and developmental regulators such as *ccrM*, *sciP*, *tacA*, *staR*, *kidO*, *spmX*, *hvyA*, *divK*, *fliX*, and *flbT*. These genes function in processes, including holdfast biosynthesis and attachment, pilus and flagellum biogenesis, cell envelope biogenesis, polysaccharide biosynthesis, cyclic-di-GMP metabolism, and polyamine transport and metabolism (31, 65–67). As CckA phosphorylates CtrA, a class I flagellar regulator (68), genes involved in flagellar assembly and chemotaxis also exhibited significantly reduced transcription (Fig. 7C and D; Fig. S7; Table S3). In contrast, genes significantly upregulated upon loss of CckA function included the nitrogen regulatory IIA protein (*CCNA_03710*) and the cell division genes *ftsL* and *mraZ*. Additionally, the (p)ppGpp-activated cell cycle regulator *mopJ* (69) and the ribosome hibernation factor *hpf* (*CCNA_03711*), which is transcriptionally activated by (p)ppGpp across diverse bacterial taxa (70–72), also showed increased expression. A small set of genes displayed minimal transcriptional changes in either single mutant [*cckA*(ts) or ∆*ntrC*] but had increased transcript levels in the double mutant, including several class II flagellar genes such as *fliP* and the *fliQ-fliR-flhB* operon (Table S3).

### *ntrC* mutation and transcriptional rescue of a subset of the CckA regulon

**Fig 8:**
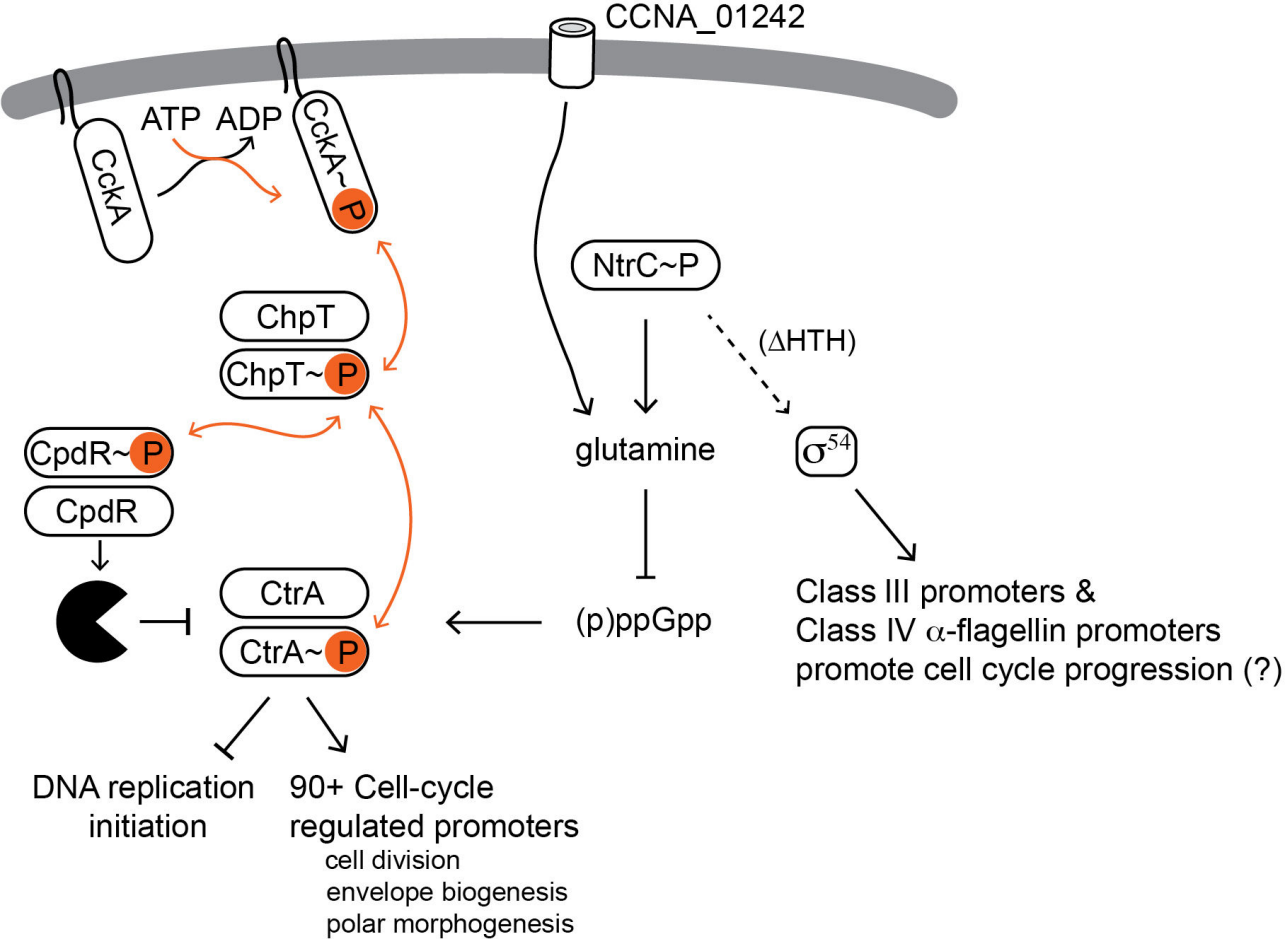
Model depicting a mechanism by which *ntrC* mutations bypass the essential function of the sensor histidine kinase, CckA. Under normal growth conditions, phosphorylated CtrA (CtrA~P) binds DNA to regulate transcription of cell cycle and cell development genes. CckA is a membrane-bound, bifunctional sensor histidine kinase that phosphorylates or dephosphorylates CtrA through the histidine phosphotransferase ChpT. Orange arrows highlight the transfer of phosphoryl groups between proteins. CpdR, a proteolytic adaptor, is also phosphorylated and dephosphorylated through ChpT. Unphosphorylated CpdR targets CtrA (and other proteins) for degradation, depicted by the black Pac-Man. Loss-of-function mutations in *ntrC* lead to reduced intracellular glutamine, which can enter the cell through the CCNA_01242 inner membrane permease. Reduced glutamine increases ppGpp levels, which are associated with sustained CtrA levels. *ntrC* mutations that disrupt binding to DNA (ΔHTH) activate transcription at non-native σ^54^-dependent promoters. We propose that this spurious activity contributes to the rescue of the defects of *cckA*(ts) at the restrictive temperature. This mechanism reveals a genetic bypass of CckA function through protein stabilization and transcriptional reprogramming.

Although the transcriptional effects of *cckA* and *ntrC* loss of function are largely independent (Fig. 7B and C), introducing *ntrC* mutations partially or fully restored the expression of a subset of genes that are dysregulated in the *cckA*(ts) mutant (Fig. 7C). To identify the genes whose transcriptional defects were more effectively rescued by *ntrC*(HTH) mutations than by *ntrC* deletion, we compared the fold change in transcript levels between *cckA*(ts)*∆ntrC* and the two point mutants, *cckA*(ts)*ntrC*(L424P) or *cckA*(ts)*ntrC*(A446P). By averaging these fold-change ratios, we observed a natural cutoff at approximately a fourfold difference, which defined a set of 24 genes whose expression was most enhanced by the HTH domain point mutations compared to *ntrC* deletion. This gene set was predominantly composed of σ^54^-regulated class III and IV flagellar genes (73–76), alongside other critical regulators, including the essential DNA methyltransferase *ccrM*—a known CtrA target (68)—and *sciP*, an inhibitor of CtrA (77, 78) (Fig. 7D; Fig. S7; Table S3). Transcription of this gene set was modestly activated relative to WT across all three single *ntrC* mutant strains, suggesting that wild-type NtrC exerts a weak repressive effect at these loci (37). In the *cckA*(ts) strain, transcription of class III and IV flagellar genes was significantly reduced, and this repression was partially alleviated by complete *ntrC* deletion [*cckA*(ts)∆*ntrC*]. However, introducing *ntrC*(HTH) point mutations into the *cckA*(ts) background led to robust activation of these σ^54^-dependent flagellar genes, which are primarily regulated by the bEBP, FlbD (65, 75). Thus, loss-of-function mutations in the DNA-binding domain of NtrC strongly enhance transcription from select σ^54^-dependent promoters, effectively rescuing gene expression defects caused by impaired CckA function (Fig. 8).

## DISCUSSION

A tightly regulated network of sensor histidine kinases and response regulators governs cell cycle progression in *Caulobacter* (66, 79, 80). The dynamics of this signaling system have been studied primarily under nutrient-replete laboratory conditions, where the levels, activities, and subcellular localization patterns of key regulatory proteins are well characterized and highly reproducible. However, *Caulobacter* cell cycle control is highly responsive to environmental and intracellular signals, including nutrient availability, redox status, and stress responses (48, 59, 64, 81–83). This raises interesting questions about how essential cell cycle sensor kinases and response regulators integrate environmental signals, and whether their functions remain indispensable under all conditions or can be bypassed when the cell is in particular physiological states. Motivated by these questions, we designed a forward selection strategy to discover genetic routes that bypass the essentiality of *cckA*, reasoning that such mutations might reveal alternative regulatory pathways influencing cell cycle control.

As expected, we identified suppressing mutations in genes that encode established components of the *Caulobacter* cell cycle control network, including second-site mutations in *cckA*, gain-of-function mutations in *ctrA*, and loss-of-function mutations in *cpdR* (Fig. 1). The strong suppression of the *cckA*(ts) phenotype in strains lacking functional *cpdR* is noteworthy and cannot be solely explained by increased stabilization of CtrA (Fig. 6), which is normally targeted for proteolysis in a CpdR-dependent manner. Data showing that another CpdR-regulated substrate, glutamate dehydrogenase (a protein involved in both glutamate catabolism and cell division) (64), contributes to suppression by *cpdR* mutation (Fig. S6) highlights the functional link between regulated proteolysis, glutamate metabolism, and cell cycle control. Although the mechanisms by which *cpdR* and *gdhZ* mutations suppress the *cckA*(ts) phenotype merit further investigation, we focused on *ntrC* mutations that rescue the growth and viability defects of a *cckA*(ts) mutant. *ntrC* encodes a bEBP best known for its conserved role in nitrogen assimilation in bacteria (84). The data presented herein reveal a functional link between *ntrC* and the essential cell cycle phosphorelay of *Caulobacter* and add to a growing body of work connecting nitrogen metabolism to cell cycle regulation (48, 81, 85–87).

### Elevated ppGpp as a mechanism for bypassing CckA

A consequence of *ntrC* loss of function is impaired glutamine biosynthesis (37). Glutamine negatively regulates (p)ppGpp synthesis (48), and our results indicate that *ntrC* mutants have increased ppGpp levels, as measured using a riboswitch-based reporter (Fig. 5A). Consistent with previous studies showing that elevated ppGpp enhances CtrA levels (59), we observed sustained CtrA protein levels in *ntrC* mutants following shutoff of gene expression. This effect is associated with bypass of CckA kinase activity and may result from enhanced CtrA stability, through reduced proteolysis, or increased translation due to mRNA stabilization. Modest increases in CtrA abundance may be sufficient to drive transcription of CtrA-regulated genes even in the absence of robust phosphorylation. This is because unphosphorylated CtrA retains the ability to bind DNA, albeit with lower affinity, a biochemical property documented in *Caulobacter* (20) and related Alphaproteobacteria, such as *Brucella* (88). These results highlight a potential mechanism by which a global stress signal can tune cell cycle progression by modulating CtrA protein levels. More generally, our results reinforce the importance of guanosine nucleotides in regulating *Caulobacter* cell cycle progression and developmental control (89, 90).

### An unexpected connection between *Caulobacter* NtrC and σ^54^-regulated gene expression

*Caulobacter* NtrC is an atypical bEBP that lacks the GAFTGA motif in its AAA+ domain (37), a structural element required for interacting with and activating σ^54^-RNA polymerase (38, 46). Our study reveals an unexpected regulatory connection between NtrC point mutants and genes activated by the σ^54^-dependent bEBP, FlbD (75). Specifically, we have shown that strains expressing *ntrC* alleles with mutations in the DNA-binding HTH domain strongly increase the levels of mRNAs transcribed from established FlbD-regulated promoters (Fig. 7; Fig. S7). Given that *Caulobacter* NtrC regulates transcription from σ^70^ promoters (37), this result raises the question of how NtrC mutants that are unable to bind their native chromosomal sites influence the levels of σ^54^-dependent transcripts. One possibility is that mutant NtrC directly interacts with FlbD-σ^54^ transcriptional complexes to activate transcription. This hypothesis is supported by studies from other systems showing that bEBPs lacking functional DNA-binding domains—whether due to C-terminal truncations or specific mutations—can still activate transcription when present at high concentrations, both *in vitro* and *in vivo* (46). Additionally, there are examples of naturally occurring bEBPs that lack C-terminal DNA-binding domains yet still activate σ^54^-dependent transcription (91).

Our data show that loss-of-function NtrC mutants have higher intracellular levels compared to wild-type NtrC (37) (Fig. 2C), which invokes a regulatory model proposed by North and Kustu (92). In this model, DNA binding primarily functions to localize bEBPs near the promoter, facilitating oligomerization and efficient activation of σ^54^-dependent transcription. However, this localization function can be bypassed when bEBPs reach sufficiently high concentrations, allowing activation to occur directly from solution. Results presented here suggest that NtrC can promote σ^54^-dependent gene expression, even without DNA-binding activity or the GAFTGA motif (Fig. 7; Fig. 8).

### NtrC mutations support the suppression of *cckA*(ts) through alternative activation mechanisms

While complete deletion of *ntrC* only partially suppresses *cckA*(ts), point mutations in its DNA-binding HTH domain more effectively restore both growth and global transcriptional profiles (Fig. 2 and 7; Fig. S4; Table S3). Notably, robust suppression by mutant NtrC requires an intact and phosphorylatable REC domain (Fig. 2E and 3), indicating that suppression is not simply due to loss of NtrC function but rather a gain of alternative regulatory activity when mutant NtrC accumulates to high concentrations and/or is no longer restricted to its native regulatory sites. This concept aligns with an early model by Magasanik (93), who proposed that DNA binding was needed to spatially constrain activation by bEBPs, preventing spurious transcription activation in bacterial genomes, which lack extensive non-coding DNA. In the case of *cckA*(ts) suppressor mutants, where bEBP expression is elevated but DNA binding is lost, mutant NtrC may acquire novel regulatory interactions with transcriptional machinery or other regulatory proteins. Additionally, changes in the levels and localization of mutant NtrC could influence the activity of other *Caulobacter* bEBPs, such as TacA (94) or FlbD (75), at their respective promoters.

Beyond direct regulatory effects, *ntrC* mutations may also impact gene expression through alterations in chromosomal architecture. NtrC binding sites overlap with those of GapR (37), a nucleoid-associated protein that affects DNA supercoiling and chromosome organization (95), which can influence cell cycle and cell development (96). Additionally, many NtrC binding sites overlap with those of MucR1 (37), a key regulator of *Caulobacter* cell cycle genes (33). Several flagellar promoters that are strongly activated in *ntrC* suppressor strains are located within chromosomal regions associated with GapR and MucR1 binding (Fig. S7). This raises the possibility that loss of NtrC DNA binding could alter chromatin organization or promote new protein-protein interactions, indirectly facilitating transcription at σ^54^-regulated sites. Although our attempts to delete *cckA* or *ctrA* in a *ntrC(*∆HTH*)* background were unsuccessful, it remains possible that the transcriptional and physiological state induced by a NtrC(HTH) mutant could permit complete deletion of *cckA* under specific conditions.

 The ability of *ntrC* mutants to bypass *cckA* function, through transcriptional reprogramming and activation of (p)ppGpp synthesis, highlights the exceptional plasticity of the *Caulobacter* cell cycle regulatory network. Our results underscore the deep integration of environmental sensing pathways with core cell cycle control systems and provide an example of how the evolution of novel gene regulatory connections can reshape cellular networks, allowing organisms to circumvent otherwise essential signaling processes.

## MATERIALS AND METHODS

### Growth conditions

*E. coli* strains were cultivated in Lysogeny Broth (LB) (10 g tryptone, 5 g yeast extract, and 10 g NaCl per L) or LB solidified with 1.5% (wt/vol) agar at 37°C. LB was supplemented with appropriate antibiotics when necessary. Antibiotic concentrations for selection of *E. coli* in solid or liquid conditions were as follows: kanamycin, 50 µg/mL (solid) and 30 µg/mL (liquid); chloramphenicol, 20 µg/mL (both); and oxytetracycline, 12 µg/mL (both). *Caulobacter* strains were cultivated in peptone yeast extract (2 g peptone, 1 g yeast extract, 1 mM MgSO_4_, and 0.5 mM CaCl_2_ per L) medium or PYE solidified with 1.5% (wt/vol) agar at 30°C or 37°C. Antibiotic concentrations for the selection of *Caulobacter* in solid and liquid conditions were as follows: kanamycin, 25 µg/mL (solid) and 5 µg/mL (liquid); chloramphenicol, 1.5 µg/mL (both); oxytetracycline, 2 µg/mL (solid) and 1 µg/mL (liquid); and gentamycin 5 µg/mL (solid) and 1 µg/mL (liquid). Nalidixic acid (20 µg/mL) was added to counterselect *E. coli* after conjugations. When noted, PYE was supplemented with an additional 9.3 mM of glutamine. When xylose was used for induction, 0.3% (wt/vol) xylose was added. For experiments in defined medium, *Caulobacter* strains were grown in M2 mineral salts with glucose (M2G) (6.1 mM Na_2_HPO_4_, 3.9 mM KH_2_PO_4_, 9.3 mM NH_4_Cl, 0.25 mM CaCl_2_, 0.5 mM MgSO_4_, 10 µM ferrous sulfate chelated with EDTA [Sigma], and 0.15% glucose). When noted, 9.3 mM NH_4_Cl was replaced with 9.3 mM glutamine.

### Strains and plasmids

Strains, plasmids, and primers used in this study are presented in Table S4. All *Caulobacter* strains are derivatives of strain NA1000 (97). To generate plasmid constructs for in-frame deletions and other allele replacements, homologous upstream and downstream fragments (~500 bp/each) were PCR-amplified and joined via overlap extension PCR (98). PCR products were cloned into plasmid pNPTS138 by restriction enzyme digestion and ligation. Similarly, to create genetic complementation constructs, target genes were amplified and fused to their upstream promoters (~500 bp fragment immediately upstream of the start of the annotated operon) via overlap extension PCR, and these fused PCR products were purified and cloned into pXGFPC-2 (pMT585) (12), which integrates into the *xylX* locus in *Caulobacter*. For complementation, genes with their native promoters were cloned in the opposite orientation of the P*xylX* promoter. For xylose-inducible expression, target genes were PCR-amplified and ligated into pMT585 in the same orientation as (i.e., downstream of) the P*xylX* promoter. To build the ppGpp reporter, the ppGpp-sensing riboswitch of *ilvE* from *D. hafniense* (54) was fused to the 5′ end of *mNeonGreen* and cloned into pXYFPC-5 (pMT604) (12), a plasmid that also integrates into the *xylX* locus in *Caulobacte*r. The riboswitch-*mNeonGreen* fusion was PCR-amplified and ligated into pXYFPC-5 in the same orientation as (i.e., downstream of) the P*xylX* promoter for xylose-inducible expression. All ligation products were transformed into *E. coli* TOP10 for propagation, and the constructs were sequence verified prior to use.

Plasmids were transformed into *Caulobacter* via electroporation or triparental mating from TOP10 using FC3 as a helper strain (99). In-frame deletion and allele replacement strains were generated via two-step recombination using *sacB* counterselection using an approach similar to that described by Fiebig and colleagues (100). Briefly, primary recombinants bearing pNPTS138-derived allele replacement plasmids were selected on solidified PYE containing kanamycin. Single colonies were then grown in PYE broth without selection for 6–18 h before secondary recombinants were selected on PYE containing 3% sucrose. The resulting clones were screened to confirm kanamycin sensitivity. Then, allele replacement was confirmed by PCR for in-frame deletion alleles or PCR amplification and Sanger sequencing for point mutation alleles.

### Selection for mutations that bypass a conditional CckA loss-of-function mutation

A mutant bearing a temperature-sensitive allele of the essential histidine kinase, CckA, was previously isolated (19). The *cckA*(ts) mutant was plated in a 10-fold dilution series on PYE medium and incubated at the restrictive temperature of 37°C. Colonies that emerged at higher dilutions (10⁻²–10⁻³) were streak purified. After confirming their ability to grow at 37°C, strains were saved in glycerol stocks. Twenty-six isolates were selected for whole-genome sequencing to identify polymorphic sites compared to the parental strain. Briefly, genomic DNA was extracted from 1 mL of saturated PYE culture using guanidinium thiocyanate, chloroform extraction, and isopropanol precipitation (101). Genomic DNA was sequenced (150 bp paired-end reads) at SeqCenter (Pittsburgh, PA, USA) using an Illumina NextSeq 2000. DNA sequencing reads were mapped to the *Caulobacter* NA1000 genome (GenBank accession CP001340) (97), and polymorphisms were identified using breseq (102).

### Serial dilution titers

Starter cultures were grown overnight at 30°C in PYE medium (Fig. 1C, 5B, and 6B; Fig. S6) or PYE supplemented with 9.3 mM glutamine (Fig. 2B, E, 3, 4A, and C; Fig. S4). Overnight cultures were diluted to an OD_660_ of 0.1 in the same medium and grown at 30°C for 2 h. After this initial outgrowth, cultures were again diluted to an OD_660_ of 0.1 and incubated in the same medium for 24 h. These stationary phase cultures were then normalized to an OD_660_ of 0.5, 10-fold serially diluted, and 5 µL of each dilution was spotted onto replicate PYE agar plates. As indicated, the agar was supplemented with 9.3 mM glutamine (PYE + gln), or with 0.3% xylose (PYE + xyl). Replicate plates were incubated at 37°C and 30°C for 4 days before imaging. Dilution plating growth experiments were performed at least three independent times for all conditions/treatments. A representative experiment is shown.

### Measurement of growth in M2G defined medium

Starter cultures were shaken overnight in PYE supplemented with 9.3 mM glutamine at 30°C. Starter cultures were pelleted and washed three times with M2G containing 9.3 mM NH_4_Cl or M2G in which NH_4_Cl was replaced with molar-equivalent glutamine before dilution to an OD_660_ of 0.025 in the respective medium. These cultures were incubated at 30°C with shaking for 24 h, and culture density was measured optically (OD_660_).

### Light microscopy

To prepare cells for imaging, starter cultures were grown in PYE overnight at 30°C and diluted to an OD_660_ of 0.1 in fresh PYE. Cultures were grown for 2 h at 30°C to allow cells to reach similar logarithmic phase growth. For Fig. 2D, logarithmic phase cultures were diluted to an OD_660_ of 0.1 in fresh PYE and grown for 3.25 h at 37°C to capture physiology at the restrictive temperature. For Fig. S3C, logarithmic phase cultures were diluted to an OD_660_ of 0.1 in fresh PYE and grown for 24 h at 30°C to allow cells to reach the stationary phase.

A volume of 2 µL of each culture was spotted on an agarose pad (1% agarose dissolved in water) on a glass slide and covered with a glass cover slip. Cells were imaged using a Leica DMI 6000 microscope using phase contrast with an HC PL APO 63×/1.4 numeric aperture oil Ph3 CS2 objective. Images were captured with an Orca-ER digital camera (Hamamatsu) controlled by Leica Application Suite X (Leica).

### RNA extraction, sequencing, and analysis

Starter cultures were grown for 18 h at 30°C in PYE. Cultures were then diluted to an OD_660_ of 0.1 in PYE and outgrown for 2 h at 30°C. Once again, cultures were diluted to an OD_660_ of 0.1 in their respective medium and grown for another 3.25 h (OD_660_ < 0.4) at 37°C to capture mRNA in logarithmic phase growth at the restrictive temperature. A volume of 6 mL of each culture was pelleted via centrifugation (1 min at 17,000 × *g*). Pellets were immediately resuspended in 1 mL TRIzol and stored at −80°C until RNA extraction. To extract RNA, thawed samples were incubated at 65°C for 10 min. After the addition of 200 µL of chloroform, samples were vortexed for 20 s and incubated at room temperature (RT) for 5 min. Phases were separated by centrifugation (10 min at 17,000 × *g*). The aqueous phase was transferred to a fresh tube, and an equal volume of isopropanol was added to precipitate the nucleic acid. Samples were stored at 80°C (1 h to overnight), then thawed and centrifuged at 17,000 × *g* for 30 min at 4°C to pellet the nucleic acid. Pellets were washed with ice-cold 70% ethanol, then centrifuged at 17,000 × *g* for 5 min at 4°C. After discarding the supernatant, pellets were air-dried at RT, resuspended in 100 µL RNase-free water, and incubated at 60°C for 10 min. Samples were treated with TURBO DNase (Invitrogen) following the manufacturer’s protocol for 30 min at RT and then column purified using RNeasy Mini Kit (Qiagen). RNA samples were sequenced at Microbial Genome Sequencing Center (Pittsburgh, PA, USA). Briefly, sequencing libraries were prepared using Illumina’s Stranded Total RNA Prep Ligation with Ribo-Zero Plus kit using custom *Caulobacter*-specific rRNA depletion probes. Fifty base pair paired-end reads were generated using the Illumina NextSeq 2000 platform (Illumina). RNA sequencing reads used to assess the impact of *ntrC* deletion on transcription have been published (37) and are available at the NCBI GEO database under series accession GSE234097. RNA sequencing reads used to measure the transcriptional impact of shifting *cckA*(ts) to the restrictive temperature and assess the effect of suppressing *ntrC* mutations are available under NCBI GEO accession GSE285684. RNA sequencing reads were mapped to the *Caulobacter* NA1000 genome (GenBank accession CP001340) (97) using default mapping parameters in CLC Genomics Workbench 22 (Qiagen), and pairwise differential gene expression analysis was performed.

We identified sets of differentially expressed genes between the ∆*ntrC* and wild-type strains, as well as between the *cckA*(ts) mutant and wild type, using stringent criteria: a maximum RPKM > 10, a fold-change threshold greater than |3|, and a false discovery rate *P*-value less than 10^−6^. Genes that were differentially regulated in either *cckA*(ts) or *ntrC* loss-of-function mutants were clustered using an uncentered correlation metric with average linkage (103), based on pairwise differential expression patterns relative to wild type. The resulting clusters were visualized using a heatmap. Notably, only a single gene overlapped between the two regulons, underscoring the distinct transcriptional responses triggered by these genetic perturbations.

To further investigate *cckA*(ts)-dysregulated genes that were more effectively restored by *ntrC* point mutations than by *ntrC* deletion, we compared pairwise differences in transcript abundance for each gene differentially expressed in the *cckA*(ts) mutant. Specifically, we evaluated:

log2[*cckA*(ts)*ntrC*(L424P)/*cckA*(ts) ∆*ntrC*],

log2[*cckA*(ts)*ntrC*(A446P)/*cckA*(ts) ∆*ntrC*].

For each gene, we calculated the average of these two comparisons to rank their relative restoration. The top 24 genes, which showed a natural break corresponding to approximately a fourfold higher expression in the presence of a *ntrC* point mutation compared to *ntrC* deletion, were selected for further analysis.

These genes were subsequently clustered based on pairwise expression differences and underwent modest manual reorganization to group genes in operons, allowing for clearer visualization of potential regulatory relationships.

### Principal component analysis of RNA-seq data

RNA-seq count data were imported from a CSV file. Normalization was performed using the counts-per-million (CPM) method to account for differences in sequencing depth across samples. The data were then log transformed [log_2_(CPM + 1)] to stabilize variance, and the log-transformed data were standardized to have a mean of zero and unit variance using the StandardScaler function. PCA was performed to reduce dimensionality and identify major sources of variation in the data set. The analysis retained three principal components, capturing the most variance in the data. The PCA results were visualized as scatterplots for the first two principal components (PC1 and PC2), which accounted for 80% of the variance. Analysis was conducted using Python, leveraging the pandas, numpy, scikit-learn, and seaborn libraries for data manipulation, PCA computation, and visualization.

### Riboswitch assay

Overnight starter cultures were grown in PYE at 30°C, diluted to an OD_660_ of 0.1 in PYE supplemented with 0.3% xylose to induce the expression of the riboswitch biosensor, and then outgrown for 2 h at 30°C. Cultures were diluted again to an OD_660_ of 0.025 in fresh PYE supplemented with 0.3% xylose and grown for 24 h at 30°C at which point green fluorescence signal from the ppGpp riboswitch fusion was measured in a Tecan Spark 20M plate reader. A volume of 200 µL of each culture was transferred to a black Costar flat, clear-bottom, 96-well plate (Corning). Cell density was measured optically at 660 nm (OD_660_), and mNeonGreen fluorescence was measured with the following wavelength parameters (excitation = 497 ± 10 nm; emission = 523 ± 10 nm). Fluorescence signal was then normalized by OD_660_.

### CtrA protein purification for antibody generation

*Caulobacter ctrA* was PCR-amplified and inserted into a pET23b-His6-SUMO expression vector using classical restriction digestion and ligation, such that *ctrA* was inserted at 3′ of the T7 promoter and the His6-SUMO coding sequence. After sequence confirmation, pET23b-His6-SUMO-*ctrA* was transformed into chemically competent *E. coli* BL21 Rosetta (DE3)/pLysS. This strain was grown in 1 L of LB at 37°C. When the culture density reached approximately OD_600_ ≈ 0.5, expression was induced with 0.5 mM isopropyl β-D-1-thiogalactopyranoside overnight at 16°C. Cells were harvested by centrifugation (10,000 × *g* for 10 min) and resuspended in 20 mL lysis buffer (20 mM Tris, pH 8, 125 mM NaCl, and 10 mM imidazole) and stored at −80°C until purification.

For protein purification, resuspended cell pellets were thawed at RT. A concentration of 1 mM phenylmethylsulfonyl fluoride was added to inhibit protease activity, and DNase I (5 µg/mL) was added to degrade DNA after cell lysis. Cells incubated on ice were lysed by sonication (Branson Instruments) at 20% magnitude for 20 s on/off pulses until the suspension was clear. The lysate was cleared of cell debris by centrifugation (30,000 × *g* for 20 min) at 4°C. The cleared lysate was applied to an affinity chromatography column containing Ni-nitrilotriacetic acid superflow resin (Qiagen) pre-equilibrated in lysis buffer. Beads were washed with a high salt wash buffer (20 mM Tris, pH 8, 500 mM NaCl, and 30 mM imidazole). Beads were then washed with a low salt wash buffer (20 mM Tris, pH 8, 1 M NaCl, and 30 mM imidazole). Protein was eluted with elution buffer (20 mM Tris, pH 8, 125 mM NaCl, and 300 mM imidazole). The elution fractions containing His6-SUMO-CtrA were pooled and dialyzed in 2 L dialysis buffer (20 mM Tris, pH 8, and 150 mM NaCl) for 4 h at 4°C to dilute the imidazole. Purified ubiquitin-like-specific protease 1 (Ulp1) was added to the eluted His6-SUMO-CtrA containing solution, which was then dialyzed overnight at 4°C in 2 L fresh dialysis buffer to cleave the His6-SUMO tag. Digested protein was mixed with 3 mL of NTA superflow resin (Qiagen) that had been pre-equilibrated in wash buffer. After incubation for 30 min at 4°C, the solution was placed onto a gravity drip column at 4°C. Flowthrough containing cleaved CtrA was collected and used to generate α-CtrA polyclonal antibodies (Pacific Immunology).

### Expression shutoff

For protein expression shutoff experiments, 25 mL of overnight PYE cultures of *Caulobacter* strains were diluted into 100 mL of fresh PYE to an OD_660_ of 0.1 and outgrown for 2 h at the permissive temperature (30°C). Cultures were then shifted to the restrictive temperature (37°C) and grown for 3.25 h before the addition of rifampicin (10 µg/mL final concentration) to inhibit transcription and, consequently, translation. After the addition of rifampicin, aliquots of 1 mL were taken at indicated time points (Fig. 6A), and cells were pelleted via centrifugation. Supernatant was discarded, and cell pellets were stored at −20°C until Western blot analysis to monitor CtrA levels, as described below.

### Western blotting

To evaluate NtrC protein levels in Fig. 2C, overnight PYE starter cultures of *Caulobacter* strains were diluted in fresh PYE to an OD_660_ of 0.1 and outgrown for 2 h at 30°C, diluted again to an OD_660_ of 0.1 in fresh PYE, and then grown for 24 h at the restrictive temperature (37°C). A 1 mL aliquot of each culture was pelleted via centrifugation. After discarding the supernatant, cell pellets were stored at −20°C until Western blot analysis.

For Western blot analysis, cell pellets were thawed and resuspended in 2× SDS loading buffer (100 mM Tris-Cl [pH 6.8], 200 mM dithiothreitol, 4% [wt/vol] SDS, 0.2% bromophenol blue, and 20% [vol/vol] glycerol) to a concentration of 0.0072 OD_660_ culture/µL loading buffer. After resuspension, genomic DNA was digested by incubation with 1 µL Benzonase per 50 µL sample volume for 20 min at RT. Samples then were denatured at 95°C for 5 min. A volume of 10 µL of each sample was loaded onto a 7.5% mini-PROTEAN precast gel (Bio-Rad) (Fig. 2C) or a 4%–20% mini-PROTEAN precast gel (Bio-Rad) (Fig. 6A) and resolved at 160–180 V at room temperature. Separated proteins were transferred from the acrylamide gel to a PVDF membrane (Millipore) using a semi-dry transfer apparatus (BioRad) at 10 V for 30 min at RT (1× Tris-Glycine and 20% methanol). Membranes were blocked in 10 mL Blotto (1× Tris-Glycine, 0.1% Tween 20 [TBST] + 5% [wt/vol] powdered milk) overnight at 4°C. The membranes were then incubated in 10 mL Blotto + polyclonal rabbit α-NtrC antiserum (1:1,000 dilution) (Fig. 2C) or polyclonal α-CtrA antiserum (1:1,000 dilution) (Fig. 6A) for 1–2 h at RT. Membranes were washed in TBST three times, 5 min per wash, before incubation in 10 mL Blotto with goat α-rabbit poly-horseradish peroxidase secondary antibody (Invitrogen; 1:10,000 dilution) for 1–2 h at RT. The membrane was then washed three times with TBST and developed with ProSignal Pico ECL Spray (Prometheus Protein Biology Products). Immediately upon spraying, the membrane was imaged using BioRad ChemiDoc Imaging System (BioRad).

## SUPPLEMENTAL MATERIAL

Supplemental Figures mbio.01962-25-s0001.pdf Figures S1-S7.

Table S1 mbio.01962-25-s0002.xlsx cckAts suppressors.

Table S2 mbio.01962-25-s0003.xlsx Glutamine mutants.

Table S3 mbio.01962-25-s0004.xlsx RNAseq data.

Table S4 mbio.01962-25-s0005.xlsx Strains.

ASM does not own the copyrights to Supplemental Material that may be linked to, or accessed through, an article. The authors have granted ASM a non-exclusive, world-wide license to publish the Supplemental Material files. Please contact the corresponding author directly for reuse.

## References

[1] Stock AM, Robinson VL, Goudreau PN. 2000. Two-component signal transduction. Annu Rev Biochem 69:183–215. doi:10.1146/annurev.biochem.69.1.183 10966457

[2] Gao R, Stock AM. 2009. Biological insights from structures of two-component proteins. Annu Rev Microbiol 63:133–154. doi:10.1146/annurev.micro.091208.073214 19575571 PMC3645274

[3] Ninfa AJ, Magasanik B. 1986. Covalent modification of the glnG product, NRI, by the glnL product, NRII, regulates the transcription of the glnALG operon in Escherichia coli. Proc Natl Acad Sci USA 83:5909–5913. doi:10.1073/pnas.83.16.5909 2874557 PMC386406

[4] Nixon BT, Ronson CW, Ausubel FM. 1986. Two-component regulatory systems responsive to environmental stimuli share strongly conserved domains with the nitrogen assimilation regulatory genes ntrB and ntrC. Proc Natl Acad Sci USA 83:7850–7854. doi:10.1073/pnas.83.20.7850 3020561 PMC386820

[5] Francis VI, Porter SL. 2019. Multikinase networks: two-component signaling networks integrating multiple stimuli. Annu Rev Microbiol 73:199–223. doi:10.1146/annurev-micro-020518-115846 31112439

[6] Zahrt TC, Deretic V. 2000. An essential two-component signal transduction system in Mycobacterium tuberculosis. J Bacteriol 182:3832–3838. doi:10.1128/JB.182.13.3832-3838.2000 10851001 PMC94557

[7] Martin PK, Li T, Sun D, Biek DP, Schmid MB. 1999. Role in cell permeability of an essential two-component system in Staphylococcus aureus. J Bacteriol 181:3666–3673. doi:10.1128/JB.181.12.3666-3673.1999 10368139 PMC93842

[8] Hecht GB, Lane T, Ohta N, Sommer JM, Newton A. 1995. An essential single domain response regulator required for normal cell division and differentiation in Caulobacter crescentus. EMBO J 14:3915–3924. doi:10.1002/j.1460-2075.1995.tb00063.x 7664732 PMC394470

[9] Fabret C, Hoch JA. 1998. A two-component signal transduction system essential for growth of Bacillus subtilis: implications for anti-infective therapy. J Bacteriol 180:6375–6383. doi:10.1128/JB.180.23.6375-6383.1998 9829949 PMC107725

[10] Boutte CC, Srinivasan BS, Flannick JA, Novak AF, Martens AT, Batzoglou S, Viollier PH, Crosson S. 2008. Genetic and computational identification of a conserved bacterial metabolic module. PLoS Genet 4:e1000310. doi:10.1371/journal.pgen.1000310 19096521 PMC2597717

[11] Stephens C, Christen B, Fuchs T, Sundaram V, Watanabe K, Jenal U. 2007. Genetic analysis of a novel pathway for D-xylose metabolism in Caulobacter crescentus. J Bacteriol 189:2181–2185. doi:10.1128/JB.01438-06 17172333 PMC1855722

[12] Thanbichler M, Iniesta AA, Shapiro L. 2007. A comprehensive set of plasmids for vanillate- and xylose-inducible gene expression in Caulobacter crescentus. Nucleic Acids Res 35:e137. doi:10.1093/nar/gkm818 17959646 PMC2175322

[13] Wilhelm RC, Singh R, Eltis LD, Mohn WW. 2019. Bacterial contributions to delignification and lignocellulose degradation in forest soils with metagenomic and quantitative stable isotope probing. ISME J 13:413–429. doi:10.1038/s41396-018-0279-6 30258172 PMC6331573

[14] Wilhelm RC. 2018. Following the terrestrial tracks of Caulobacter - redefining the ecology of a reputed aquatic oligotroph. ISME J 12:3025–3037. doi:10.1038/s41396-018-0257-z 30108303 PMC6246563

[15] Zik JJ, Ryan KR. 2022. Cell cycle signal transduction and proteolysis in *Caulobacter*. In Biondi EG (ed), Cell cycle regulation and development in alphaproteobacteria. Springer.

[16] Biondi EG, Reisinger SJ, Skerker JM, Arif M, Perchuk BS, Ryan KR, Laub MT. 2006. Regulation of the bacterial cell cycle by an integrated genetic circuit. Nature 444:899–904. doi:10.1038/nature05321 17136100

[17] Chen YE, Tsokos CG, Biondi EG, Perchuk BS, Laub MT. 2009. Dynamics of two phosphorelays controlling cell cycle progression in Caulobacter crescentus. J Bacteriol 191:7417–7429. doi:10.1128/JB.00992-09 19783630 PMC2786585

[18] Jacobs C, Ausmees N, Cordwell SJ, Shapiro L, Laub MT. 2003. Functions of the CckA histidine kinase in Caulobacter cell cycle control. Mol Microbiol 47:1279–1290. doi:10.1046/j.1365-2958.2003.03379.x 12603734

[19] Jacobs C, Domian IJ, Maddock JR, Shapiro L. 1999. Cell cycle-dependent polar localization of an essential bacterial histidine kinase that controls DNA replication and cell division. Cell 97:111–120. doi:10.1016/s0092-8674(00)80719-9 10199407

[20] Siam R, Marczynski GT. 2000. Cell cycle regulator phosphorylation stimulates two distinct modes of binding at a chromosome replication origin. EMBO J 19:1138–1147. doi:10.1093/emboj/19.5.1138 10698954 PMC305652

[21] Laub MT, McAdams HH, Feldblyum T, Fraser CM, Shapiro L. 2000. Global analysis of the genetic network controlling a bacterial cell cycle. Science 290:2144–2148. doi:10.1126/science.290.5499.2144 11118148

[22] Ahmed YM, Brown LM, Varga K, Bowman GR. 2024. Phospho-signaling couples polar asymmetry and proteolysis within a membraneless microdomain in Caulobacter crescentus. Nat Commun 15:9282. doi:10.1038/s41467-024-53395-y 39468040 PMC11519897

[23] Iniesta AA, McGrath PT, Reisenauer A, McAdams HH, Shapiro L. 2006. A phospho-signaling pathway controls the localization and activity of a protease complex critical for bacterial cell cycle progression. Proc Natl Acad Sci USA 103:10935–10940. doi:10.1073/pnas.0604554103 16829582 PMC1544152

[24] Lau J, Hernandez-Alicea L, Vass RH, Chien P. 2015. A phosphosignaling adaptor primes the AAA+ protease ClpXP to drive cell cycle-regulated proteolysis. Mol Cell 59:104–116. doi:10.1016/j.molcel.2015.05.014 26073542 PMC4490964

[25] Lori C, Ozaki S, Steiner S, Böhm R, Abel S, Dubey BN, Schirmer T, Hiller S, Jenal U. 2015. Cyclic di-GMP acts as a cell cycle oscillator to drive chromosome replication. Nature 523:236–239. doi:10.1038/nature14473 25945741

[26] Dubey BN, Lori C, Ozaki S, Fucile G, Plaza-Menacho I, Jenal U, Schirmer T. 2016. Cyclic di-GMP mediates a histidine kinase/phosphatase switch by noncovalent domain cross-linking. Sci Adv 2:e1600823. doi:10.1126/sciadv.1600823 27652341 PMC5026420

[27] Mann Thomas H, Seth Childers W, Blair JA, Eckart MR, Shapiro L. 2016. A cell cycle kinase with tandem sensory PAS domains integrates cell fate cues. Nat Commun 7:11454. doi:10.1038/ncomms11454 27117914 PMC4853435

[28] Mann T.H, Shapiro L. 2018. Integration of cell cycle signals by multi-PAS domain kinases. Proc Natl Acad Sci USA 115:E7166–E7173. doi:10.1073/pnas.1808543115 29987042 PMC6065003

[29] Heinrich K, Sobetzko P, Jonas K. 2016. A kinase-phosphatase switch transduces environmental information into a bacterial cell cycle circuit. PLoS Genet 12:e1006522. doi:10.1371/journal.pgen.1006522 27941972 PMC5189948

[30] Kirkpatrick CL, Viollier PH. 2012. Decoding Caulobacter development. FEMS Microbiol Rev 36:193–205. doi:10.1111/j.1574-6976.2011.00309.x 22091823

[31] Lasker K, Mann TH, Shapiro L. 2016. An intracellular compass spatially coordinates cell cycle modules in Caulobacter crescentus. Curr Opin Microbiol 33:131–139. doi:10.1016/j.mib.2016.06.007 27517351 PMC5069156

[32] Tsokos CG, Perchuk BS, Laub MT. 2011. A dynamic complex of signaling proteins uses polar localization to regulate cell-fate asymmetry in Caulobacter crescentus. Dev Cell 20:329–341. doi:10.1016/j.devcel.2011.01.007 21397844 PMC3068846

[33] Fumeaux C, Radhakrishnan SK, Ardissone S, Théraulaz L, Frandi A, Martins D, Nesper J, Abel S, Jenal U, Viollier PH. 2014. Cell cycle transition from S-phase to G1 in Caulobacter is mediated by ancestral virulence regulators. Nat Commun 5:4081. doi:10.1038/ncomms5081 24939058 PMC4083442

[34] Hirschman J, Wong PK, Sei K, Keener J, Kustu S. 1985. Products of nitrogen regulatory genes ntrA and ntrC of enteric bacteria activate glnA transcription in vitro: evidence that the ntrA product is a sigma factor. Proc Natl Acad Sci USA 82:7525–7529. doi:10.1073/pnas.82.22.7525 2999766 PMC390849

[35] Hunt TP, Magasanik B. 1985. Transcription of glnA by purified Escherichia coli components: core RNA polymerase and the products of glnF, glnG, and glnL. Proc Natl Acad Sci USA 82:8453–8457. doi:10.1073/pnas.82.24.8453 2867543 PMC390934

[36] Wong PK, Popham D, Keener J, Kustu S. 1987. In vitro transcription of the nitrogen fixation regulatory operon nifLA of Klebsiella pneumoniae. J Bacteriol 169:2876–2880. doi:10.1128/jb.169.6.2876-2880.1987 3294810 PMC212204

[37] North H, McLaughlin M, Fiebig A, Crosson S. 2023. The Caulobacter NtrB-NtrC two-component system bridges nitrogen assimilation and cell development. J Bacteriol 205:e0018123. doi:10.1128/jb.00181-23 37791753 PMC10601693

[38] Dago AE, Wigneshweraraj SR, Buck M, Morett E. 2007. A role for the conserved GAFTGA motif of AAA+ transcription activators in sensing promoter dna conformation. J Biol Chem 282:1087–1097. doi:10.1074/jbc.M608715200 17090527

[39] Domian IJ, Quon KC, Shapiro L. 1997. Cell type-specific phosphorylation and proteolysis of a transcriptional regulator controls the G1-to-S transition in a bacterial cell cycle. Cell 90:415–424. doi:10.1016/s0092-8674(00)80502-4 9267022

[40] Iniesta AA, Shapiro L. 2008. A bacterial control circuit integrates polar localization and proteolysis of key regulatory proteins with a phospho-signaling cascade. Proc Natl Acad Sci USA 105:16602–16607. doi:10.1073/pnas.0808807105 18946044 PMC2575466

[41] Delaby M, Panis G, Viollier PH. 2019. Bacterial cell cycle and growth phase switch by the essential transcriptional regulator CtrA. Nucleic Acids Res 47:10628–10644. doi:10.1093/nar/gkz846 31598724 PMC6847485

[42] Vidangos NK, Heideker J, Lyubimov A, Lamers M, Huo Y, Pelton JG, Ton J, Gralla J, Berger J, Wemmer DE. 2014. DNA recognition by a σ(54) transcriptional activator from Aquifex aeolicus. J Mol Biol 426:3553–3568. doi:10.1016/j.jmb.2014.08.009 25158097 PMC4188747

[43] Chakrabartty A, Kortemme T, Baldwin RL. 1994. Helix propensities of the amino acids measured in alanine-based peptides without helix-stabilizing side-chain interactions. Protein Sci 3:843–852. doi:10.1002/pro.5560030514 8061613 PMC2142718

[44] O’Neil KT, DeGrado WF. 1990. A thermodynamic scale for the helix-forming tendencies of the commonly occurring amino acids. Science 250:646–651. doi:10.1126/science.2237415 2237415

[45] Bowman WC, Kranz RG. 1998. A bacterial ATP-dependent, enhancer binding protein that activates the housekeeping RNA polymerase. Genes Dev 12:1884–1893. doi:10.1101/gad.12.12.1884 9637689 PMC316913

[46] Bush M, Dixon R. 2012. The role of bacterial enhancer binding proteins as specialized activators of σ54-dependent transcription. Microbiol Mol Biol Rev 76:497–529. doi:10.1128/MMBR.00006-12 22933558 PMC3429621

[47] Hwang I, Thorgeirsson T, Lee J, Kustu S, Shin YK. 1999. Physical evidence for a phosphorylation-dependent conformational change in the enhancer-binding protein NtrC. Proc Natl Acad Sci USA 96:4880–4885. doi:10.1073/pnas.96.9.4880 10220387 PMC21785

[48] Ronneau S, Petit K, De Bolle X, Hallez R. 2016. Phosphotransferase-dependent accumulation of (p)ppGpp in response to glutamine deprivation in Caulobacter crescentus. Nat Commun 7:11423. doi:10.1038/ncomms11423 27109061 PMC4848567

[49] Poindexter JS. 1981. Oligotrophy: fast and famine existence. Adv Microb Ecol 5:63–89. doi:10.1007/978-1-4615-8306-6_2

[50] Hershey DM, Fiebig A, Crosson S. 2019. A genome-wide analysis of adhesion in Caulobacter crescentus identifies new regulatory and biosynthetic components for holdfast assembly. mBio 10:e02273-18. doi:10.1128/mBio.02273-18 30755507 PMC6372794

[51] Price MN, Wetmore KM, Waters RJ, Callaghan M, Ray J, Liu H, Kuehl JV, Melnyk RA, Lamson JS, Suh Y, Carlson HK, Esquivel Z, Sadeeshkumar H, Chakraborty R, Zane GM, Rubin BE, Wall JD, Visel A, Bristow J, Blow MJ, Arkin AP, Deutschbauer AM. 2018. Mutant phenotypes for thousands of bacterial genes of unknown function. Nature 557:503–509. doi:10.1038/s41586-018-0124-0 29769716

[52] Boutte CC, Crosson S. 2013. Bacterial lifestyle shapes stringent response activation. Trends Microbiol 21:174–180. doi:10.1016/j.tim.2013.01.002 23419217 PMC4238387

[53] Dworkin J, Harwood CS. 2022. Metabolic reprogramming and longevity in quiescence. Annu Rev Microbiol 76:91–111. doi:10.1146/annurev-micro-041320-111014 35417196

[54] Sherlock ME, Sudarsan N, Breaker RR. 2018. Riboswitches for the alarmone ppGpp expand the collection of RNA-based signaling systems. Proc Natl Acad Sci USA 115:6052–6057. doi:10.1073/pnas.1720406115 29784782 PMC6003355

[55] Shaner NC, Lambert GG, Chammas A, Ni Y, Cranfill PJ, Baird MA, Sell BR, Allen JR, Day RN, Israelsson M, Davidson MW, Wang J. 2013. A bright monomeric green fluorescent protein derived from Branchiostoma lanceolatum. Nat Methods 10:407–409. doi:10.1038/nmeth.2413 23524392 PMC3811051

[56] Sun Z, Wu R, Zhao B, Zeinert R, Chien P, You M. 2021. Live‐cell imaging of guanosine tetra‐ and pentaphosphate (p)ppGpp with RNA‐based fluorescent sensors**. Angew Chem Int Ed 60:24070–24074. doi:10.1002/anie.202111170 PMC8545912 34487413

[57] Hydorn M, Nagarajan SN, Fones E, Harwood CS, Dworkin J. 2025. Analysis of (p)ppGpp metabolism and signaling using a dynamic luminescent reporter. bioRxiv. doi:10.1101/2025.04.16.649062 PMC12373219 40845056

[58] Boutte CC, Crosson S. 2011. The complex logic of stringent response regulation in Caulobacter crescentus: starvation signalling in an oligotrophic environment. Mol Microbiol 80:695–714. doi:10.1111/j.1365-2958.2011.07602.x 21338423 PMC3093662

[59] Gonzalez D, Collier J. 2014. Effects of (p)ppGpp on the progression of the cell cycle of Caulobacter crescentus. J Bacteriol 196:2514–2525. doi:10.1128/JB.01575-14 24794566 PMC4097592

[60] Schreiber G, Metzger S, Aizenman E, Roza S, Cashel M, Glaser G. 1991. Overexpression of the relA gene in Escherichia coli. J Biol Chem 266:3760–3767. 1899866

[61] Joshi KK, Bergé M, Radhakrishnan SK, Viollier PH, Chien P. 2015. An adaptor hierarchy regulates proteolysis during a bacterial cell cycle. Cell 163:419–431. doi:10.1016/j.cell.2015.09.030 26451486 PMC4600535

[62] Ryan KR, Judd EM, Shapiro L. 2002. The CtrA response regulator essential for Caulobacter crescentus cell-cycle progression requires a bipartite degradation signal for temporally controlled proteolysis. J Mol Biol 324:443–455. doi:10.1016/s0022-2836(02)01042-2 12445780

[63] Radhakrishnan SK, Pritchard S, Viollier PH. 2010. Coupling prokaryotic cell fate and division control with a bifunctional and oscillating oxidoreductase homolog. Dev Cell 18:90–101. doi:10.1016/j.devcel.2009.10.024 20152180

[64] Beaufay F, Coppine J, Mayard A, Laloux G, De Bolle X, Hallez R. 2015. A NAD-dependent glutamate dehydrogenase coordinates metabolism with cell division in Caulobacter crescentus. EMBO J 34:1786–1800. doi:10.15252/embj.201490730 25953831 PMC4516431

[65] Ardissone S, Viollier PH. 2015. Interplay between flagellation and cell cycle control in Caulobacter. Curr Opin Microbiol 28:83–92. doi:10.1016/j.mib.2015.08.012 26476805

[66] Barrows JM, Goley ED. 2023. Synchronized swarmers and sticky stalks: Caulobacter crescentus as a model for bacterial cell biology. J Bacteriol 205:e0038422. doi:10.1128/jb.00384-22 36715542 PMC9945503

[67] Curtis PD, Brun YV. 2010. Getting in the loop: regulation of development in Caulobacter crescentus. Microbiol Mol Biol Rev 74:13–41. doi:10.1128/MMBR.00040-09 20197497 PMC2832345

[68] Quon KC, Marczynski GT, Shapiro L. 1996. Cell cycle control by an essential bacterial two-component signal transduction protein. Cell 84:83–93. doi:10.1016/s0092-8674(00)80995-2 8548829

[69] Sanselicio S, Bergé M, Théraulaz L, Radhakrishnan SK, Viollier PH. 2015. Topological control of the Caulobacter cell cycle circuitry by a polarized single-domain PAS protein. Nat Commun 6:7005. doi:10.1038/ncomms8005 25952018 PMC4432633

[70] Hood RD, Higgins SA, Flamholz A, Nichols RJ, Savage DF. 2016. The stringent response regulates adaptation to darkness in the Cyanobacterium Synechococcus elongatus. Proc Natl Acad Sci USA 113:E4867–E4876. doi:10.1073/pnas.1524915113 27486247 PMC4995992

[71] Izutsu K, Wada A, Wada C. 2001. Expression of ribosome modulation factor (RMF) in Escherichia coli requires ppGpp. Genes Cells 6:665–676. doi:10.1046/j.1365-2443.2001.00457.x 11532026

[72] Schäfer H, Beckert B, Frese CK, Steinchen W, Nuss AM, Beckstette M, Hantke I, Driller K, Sudzinová P, Krásný L, Kaever V, Dersch P, Bange G, Wilson DN, Turgay K. 2020. The alarmones (p)ppGpp are part of the heat shock response of Bacillus subtilis. PLoS Genet 16:e1008275. doi:10.1371/journal.pgen.1008275 32176689 PMC7098656

[73] Benson AK, Wu J, Newton A. 1994. The role of FlbD in regulation of flagellar gene transcription in Caulobacter crescentus. Res Microbiol 145:420–430. doi:10.1016/0923-2508(94)90090-6 7855428

[74] Newton A, Ohta N, Ramakrishnan G, Mullin D, Raymond G. 1989. Genetic switching in the flagellar gene hierarchy of Caulobacter requires negative as well as positive regulation of transcription. Proc Natl Acad Sci USA 86:6651–6655. doi:10.1073/pnas.86.17.6651 2771949 PMC297903

[75] Wingrove JA, Gober JW. 1994. A sigma 54 transcriptional activator also functions as a pole-specific repressor in Caulobacter. Genes Dev 8:1839–1852. doi:10.1101/gad.8.15.1839 7958861

[76] Xu H, Dingwall A, Shapiro L. 1989. Negative transcriptional regulation in the Caulobacter flagellar hierarchy. Proc Natl Acad Sci USA 86:6656–6660. doi:10.1073/pnas.86.17.6656 2771950 PMC297904

[77] Gora KG, Tsokos CG, Chen YE, Srinivasan BS, Perchuk BS, Laub MT. 2010. A cell-type-specific protein-protein interaction modulates transcriptional activity of a master regulator in Caulobacter crescentus. Mol Cell 39:455–467. doi:10.1016/j.molcel.2010.06.024 20598601 PMC3073018

[78] Tan MH, Kozdon JB, Shen X, Shapiro L, McAdams HH. 2010. An essential transcription factor, SciP, enhances robustness of Caulobacter cell cycle regulation. Proc Natl Acad Sci USA 107:18985–18990. doi:10.1073/pnas.1014395107 20956288 PMC2973855

[79] Collier J. 2019. Cell division control in Caulobacter crescentus. Biochim Biophys Acta Gene Regul Mech 1862:685–690. doi:10.1016/j.bbagrm.2018.04.005 29715525

[80] van Teeseling MCF, Thanbichler M. 2020. Generating asymmetry in a changing environment: cell cycle regulation in dimorphic alphaproteobacteria. Biol Chem 401:1349–1363. doi:10.1515/hsz-2020-0235 32970604

[81] Boutte CC, Henry JT, Crosson S. 2012. ppGpp and polyphosphate modulate cell cycle progression in Caulobacter crescentus. J Bacteriol 194:28–35. doi:10.1128/JB.05932-11 22020649 PMC3256613

[82] Hallgren J, Koonce K, Felletti M, Mortier J, Turco E, Jonas K. 2023. Phosphate starvation decouples cell differentiation from DNA replication control in the dimorphic bacterium Caulobacter crescentus. PLoS Genet 19:e1010882. doi:10.1371/journal.pgen.1010882 38011258 PMC10723716

[83] Narayanan S, Janakiraman B, Kumar L, Radhakrishnan SK. 2015. A cell cycle-controlled redox switch regulates the topoisomerase IV activity. Genes Dev 29:1175–1187. doi:10.1101/gad.257030.114 26063575 PMC4470285

[84] Stewart V. 1994. Regulation of nitrate and nitrite reductase synthesis in Enterobacteria. Antonie Van Leeuwenhoek 66:37–45. doi:10.1007/BF00871631 7747939

[85] Chiaverotti TA, Parker G, Gallant J, Agabian N. 1981. Conditions that trigger guanosine tetraphosphate accumulation in Caulobacter crescentus. J Bacteriol 145:1463–1465. doi:10.1128/jb.145.3.1463-1465.1981 7204347 PMC217160

[86] England JC, Perchuk BS, Laub MT, Gober JW. 2010. Global regulation of gene expression and cell differentiation in Caulobacter crescentus in response to nutrient availability. J Bacteriol 192:819–833. doi:10.1128/JB.01240-09 19948804 PMC2812448

[87] Xu C, Weston BR, Tyson JJ, Cao Y. 2020. Cell cycle control and environmental response by second messengers in Caulobacter crescentus. BMC Bioinformatics 21:408. doi:10.1186/s12859-020-03687-z 32998723 PMC7526171

[88] Herrou J, Willett JW, Fiebig A, Varesio LM, Czyż DM, Cheng JX, Ultee E, Briegel A, Bigelow L, Babnigg G, Kim Y, Crosson S. 2019. Periplasmic protein EipA determines envelope stress resistance and virulence in Brucella abortus. Mol Microbiol 111:637–661. doi:10.1111/mmi.14178 30536925 PMC6417958

[89] Glenn S, Fragasso A, Lin WH, Papagiannakis A, Kato S, Jacobs-Wagner C. 2024. Coupling of cell growth modulation to asymmetric division and cell cycle regulation in Caulobacter crescentus Proc Natl Acad Sci USA 121:e2406397121. doi:10.1073/pnas.2406397121 39361646 PMC11474046

[90] Hallez R, Delaby M, Sanselicio S, Viollier PH. 2017. Hit the right spots: cell cycle control by phosphorylated guanosines in alphaproteobacteria. Nat Rev Microbiol 15:137–148. doi:10.1038/nrmicro.2016.183 28138140

[91] Beck LL, Smith TG, Hoover TR. 2007. Look, no hands! unconventional transcriptional activators in bacteria. Trends Microbiol 15:530–537. doi:10.1016/j.tim.2007.09.008 17997097

[92] North AK, Kustu S. 1997. Mutant forms of the enhancer-binding protein NtrC can activate transcription from solution. J Mol Biol 267:17–36. doi:10.1006/jmbi.1996.0838 9096204

[93] Magasanik B. 1989. Gene regulation from sites near and far. New Biol 1:247–251. 2487290

[94] Biondi EG, Skerker JM, Arif M, Prasol MS, Perchuk BS, Laub MT. 2006. A phosphorelay system controls stalk biogenesis during cell cycle progression in Caulobacter crescentus. Mol Microbiol 59:386–401. doi:10.1111/j.1365-2958.2005.04970.x 16390437

[95] Ricci DP, Melfi MD, Lasker K, Dill DL, McAdams HH, Shapiro L. 2016. Cell cycle progression in Caulobacter requires a nucleoid-associated protein with high AT sequence recognition. Proc Natl Acad Sci USA 113:E5952–E5961. doi:10.1073/pnas.1612579113 27647925 PMC5056096

[96] Guo MS, Haakonsen DL, Zeng W, Schumacher MA, Laub MT. 2018. A bacterial chromosome structuring protein binds overtwisted dna to stimulate type ii topoisomerases and enable dna replication. Cell 175:583–597. doi:10.1016/j.cell.2018.08.029 30220456 PMC6173638

[97] Marks ME, Castro-Rojas CM, Teiling C, Du L, Kapatral V, Walunas TL, Crosson S. 2010. The genetic basis of laboratory adaptation in Caulobacter crescentus. J Bacteriol 192:3678–3688. doi:10.1128/JB.00255-10 20472802 PMC2897358

[98] Ho SN, Hunt HD, Horton RM, Pullen JK, Pease LR. 1989. Site-directed mutagenesis by overlap extension using the polymerase chain reaction. Gene 77:51–59. doi:10.1016/0378-1119(89)90358-2 2744487

[99] Ely B. 1991. Genetics of Caulobacter crescentus. Methods Enzymol 204:372–384. doi:10.1016/0076-6879(91)04019-k 1658564

[100] Fiebig A, Castro Rojas CM, Siegal-Gaskins D, Crosson S. 2010. Interaction specificity, toxicity and regulation of a paralogous set of ParE/RelE-family toxin-antitoxin systems. Mol Microbiol 77:236–251. doi:10.1111/j.1365-2958.2010.07207.x 20487277 PMC2907451

[101] Pitcher DG, Saunders NA, Owen RJ. 1989. Rapid extraction of bacterial genomic DNA with guanidium thiocyanate. Lett Appl Microbiol 8:151–156. doi:10.1111/j.1472-765X.1989.tb00262.x

[102] Deatherage DE, Barrick JE. 2014. Identification of mutations in laboratory-evolved microbes from next-generation sequencing data using breseq. Methods Mol Biol 1151:165–188. doi:10.1007/978-1-4939-0554-6_12 24838886 PMC4239701

[103] de Hoon MJL, Imoto S, Nolan J, Miyano S. 2004. Open source clustering software. Bioinformatics 20:1453–1454. doi:10.1093/bioinformatics/bth078 14871861

