# Supplementary material for: Disrupting NtrC function reveals unexpected robustness in a central cell cycle regulatory network
Source: mBio. 2025 Aug 18;16(9):e01962-25. doi: 10.1128/mbio.01962-25 (PMC12421884; doi:10.1128/mbio.01962-25)
Supplement: Supplemental Figures — Figures S1-S7. [file mbio.01962-25-s0001.pdf]

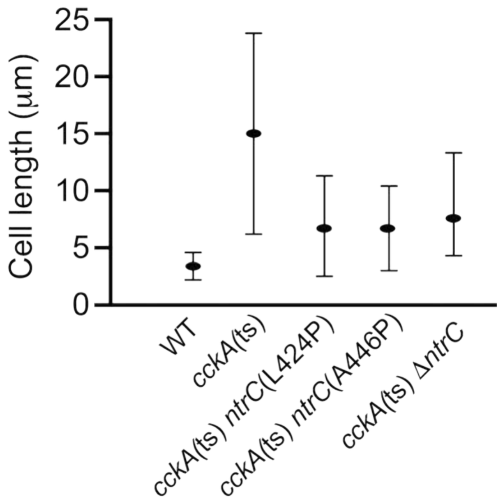

Figure S1. **Quantification of cell length from phase contrast light micrographs**. WT (n=372), *cckA*(ts) (n=40), and *ntrC* mutant suppressor strains (n=641, 340, & 428, from right to left) grown at the restrictive temperature (37°C) for 3.25 hours are shown. Cell length data represent median ± standard deviation and were quantified using BacStalk [1].

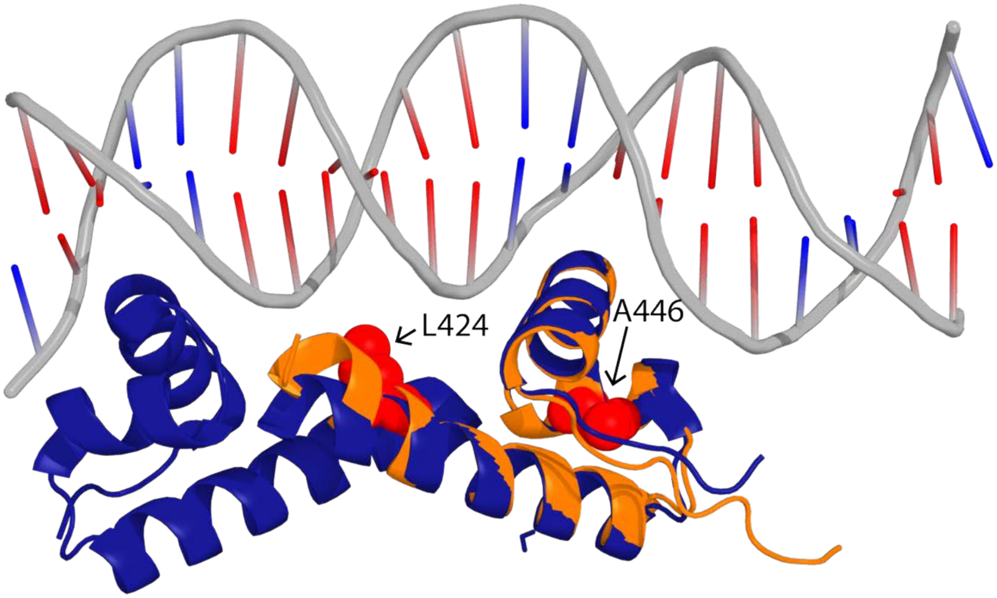

Figure S2. Structural overlay of *Caulobacter* and *Aquifex* NtrC HTH domains. The *Caulobacter* NtrC helix-turn-helix (HTH) domain (Uniprot AlphaFold prediction; orange) is superimposed on chain A of an experimental crystal structure of the *Aquifex aeolicus* NtrC HTH domain bound to DNA (PDB: 4FTH; navy blue) [2] using the `super` function in PyMol [3]. The HTH binds as a dimer in the experimental structure; Chain B of the *A. aeolicus* HTH domain is also shown (navy blue; left of chain A). Residues corresponding to the suppressor mutations L424P and A446P in *Caulobacter* NtrC are shown as red spheres and highlighted with arrows. The DNA backbone from the 4FTH structure is rendered in gray.

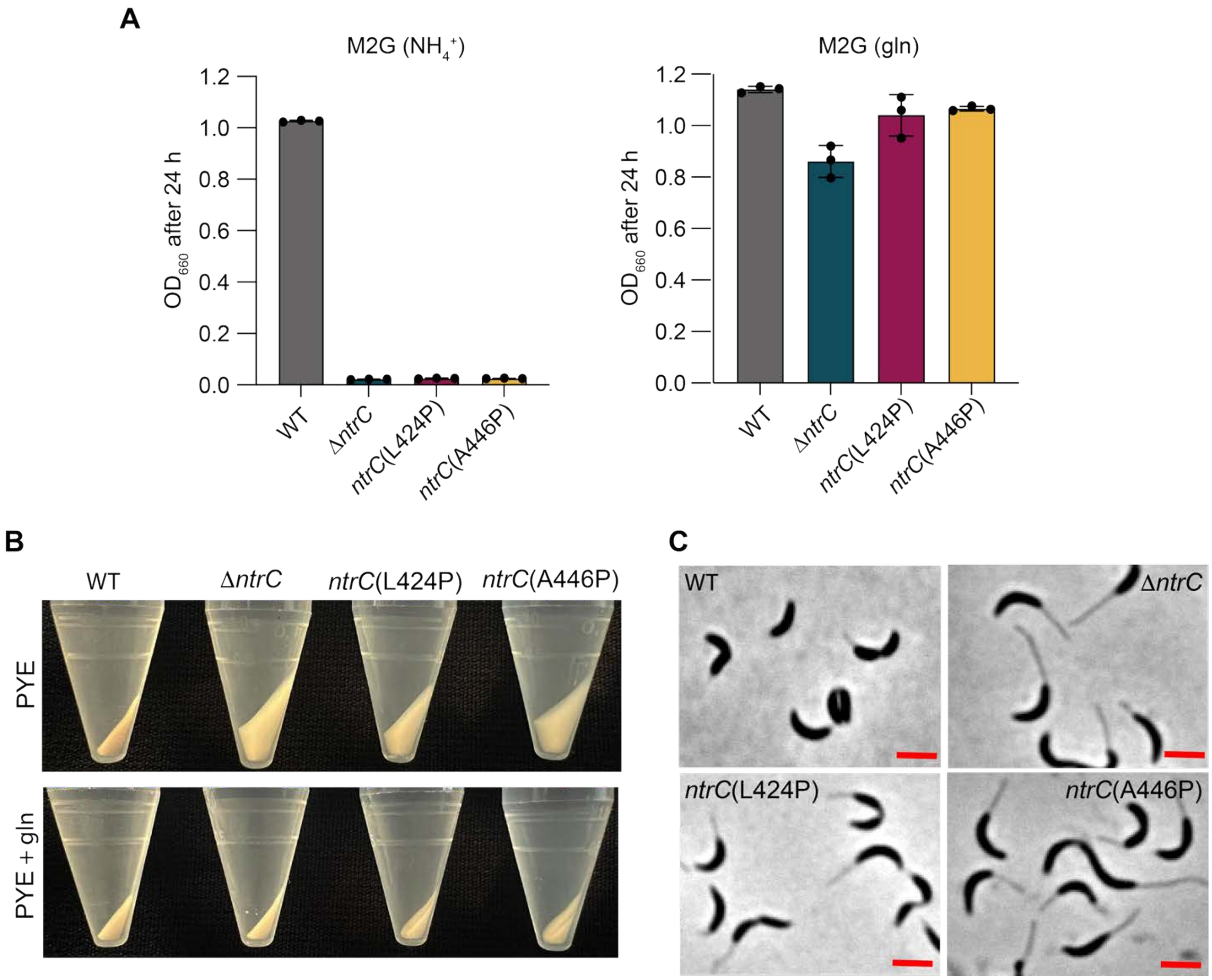

Figure S3. NtrC(HTH) point mutants phenocopy Δ*ntrC* in otherwise wild-type background of *Caulobacter*. (A) Terminal culture densities of WT, Δ*ntrC*, *ntrC*(L424P), and *ntrC*(A446P). Culture growth was measured spectrophotometrically at 660 nm (OD_{660}) after 24 hours (h) of growth in defined M2G or M2G in which NH_4^+ was replaced with molar-equivalent (9.3 mM final concentration) glutamine (gln). Data represent the mean ± standard deviation of three biological replicates. (B) Cell pellets of WT, Δ*ntrC*, *ntrC*(L424P), and *ntrC*(A446P) strains highlighting differences in pellet density. Strains were grown overnight in PYE complex medium or PYE supplemented with 9.3 mM gln, as indicated. Overnight cultures were normalized to OD_{660} 0.5 and 10 ml of each culture was centrifuged at 7,197 × *g* for 3 min at 4°C to pellet the cells. (C) Representative phase contrast images of cell stalks from WT, Δ*ntrC*, *ntrC*(L424P), and *ntrC*(A446P) strains. Images were taken after 24 h of growth in PYE broth to capture cells in stationary phase. Scale bar (red, bottom right) equals 5 μm.

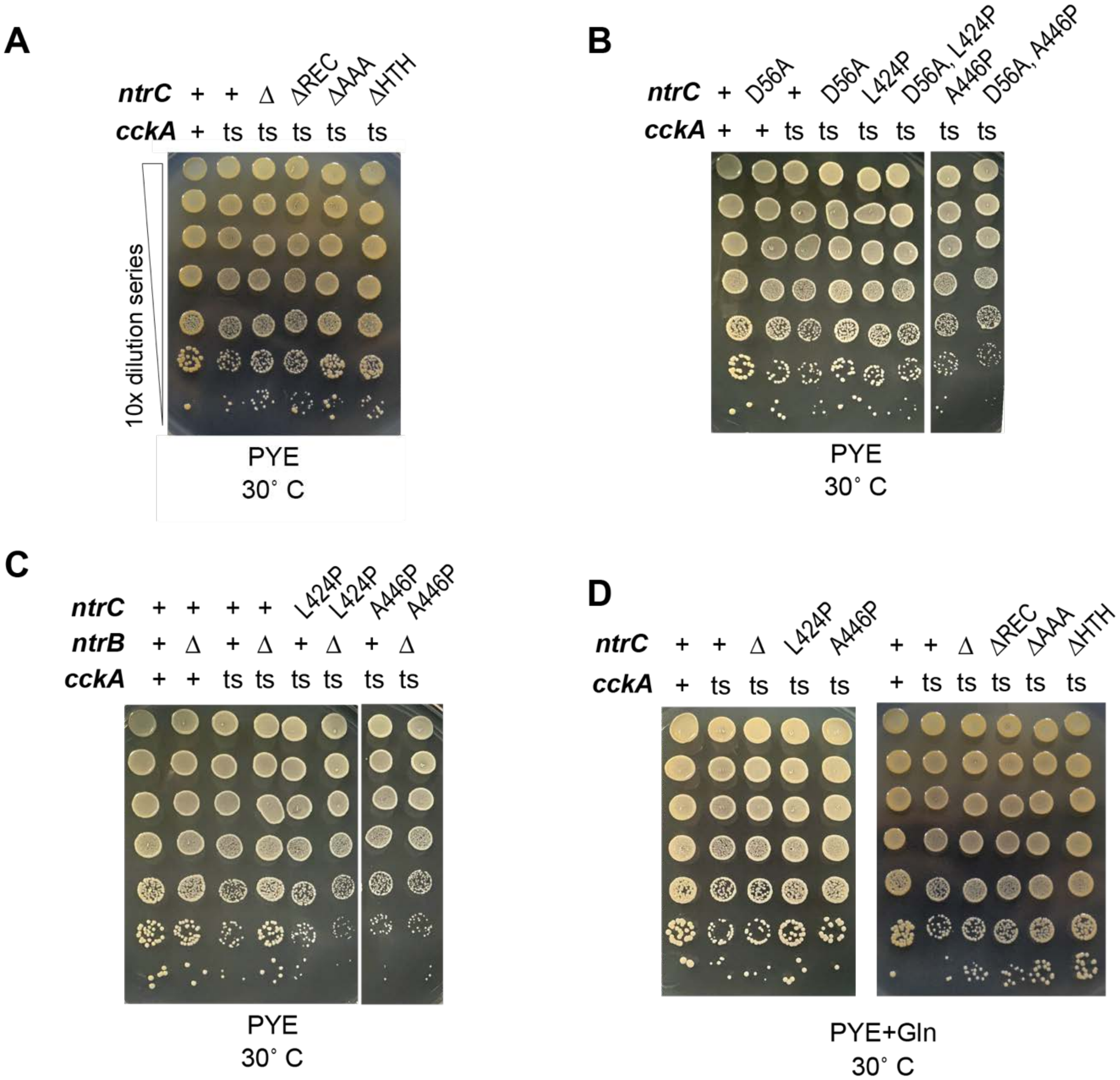

Figure S4. Permissive temperature control titers. Control (30˚C) titers corresponding to the following main text figure panels: (A) Figure 2E, (B) Figure 3A, (C) Figure 3B, and (D) Figure 4A. All dilution series were spotted onto PYE or PYE supplemented with an additional 9.3 mM glutamine (gln) and incubated for four days at 30°C before imaging.

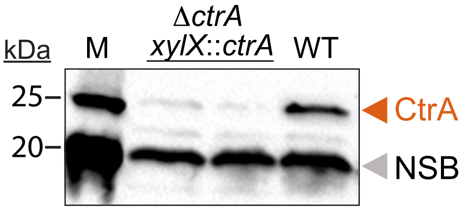

Figure S5. Immunoblot of wild type (WT) and replicate cultures of a conditional *ctrA* depletion strain (Δ*ctrA xylX*::*ctrA*) probed with anti-CtrA serum confirms the CtrA band. M = molecular weight standards. NSB = non-specific band that reacts with polyclonal CtrA antiserum.

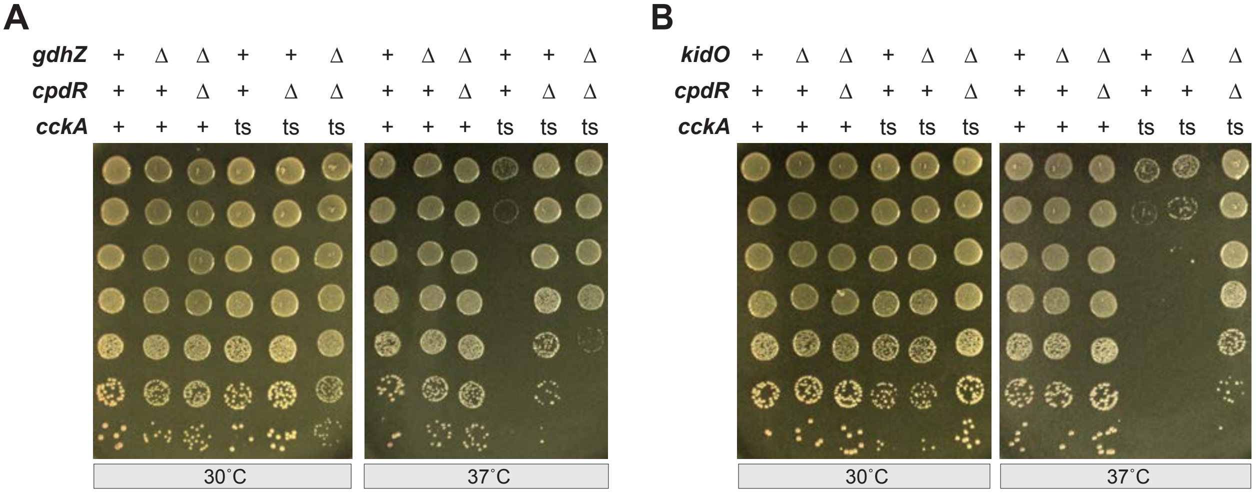

Figure S6. Assessing the impacts of *kidO* or *gdhZ* deletion on suppression of the *cckA*(ts) phenotype by Δ*cpdR*. Serial dilution of *Caulobacter* strains encoding wild-type (+), or the temperature sensitive (ts) allele of *cckA*, and/or in-frame deletion alleles (Δ) of *cpdR*, *gdhZ* or *kidO*, grown for four days at the permissive (30˚C) or restrictive (37˚C) temperature. At the restrictive temperature, deletion of the proteolytic adapter, *cpdR*, fully rescues growth of the *cckA*(ts) strain. (A) shows the impact of *gdhZ* deletion on Δ*cpdR* rescue of the *cckA*(ts) phenotype. (B) shows the impact of *kidO* deletion on Δ*cpdR* rescue of the *cckA*(ts) phenotype.

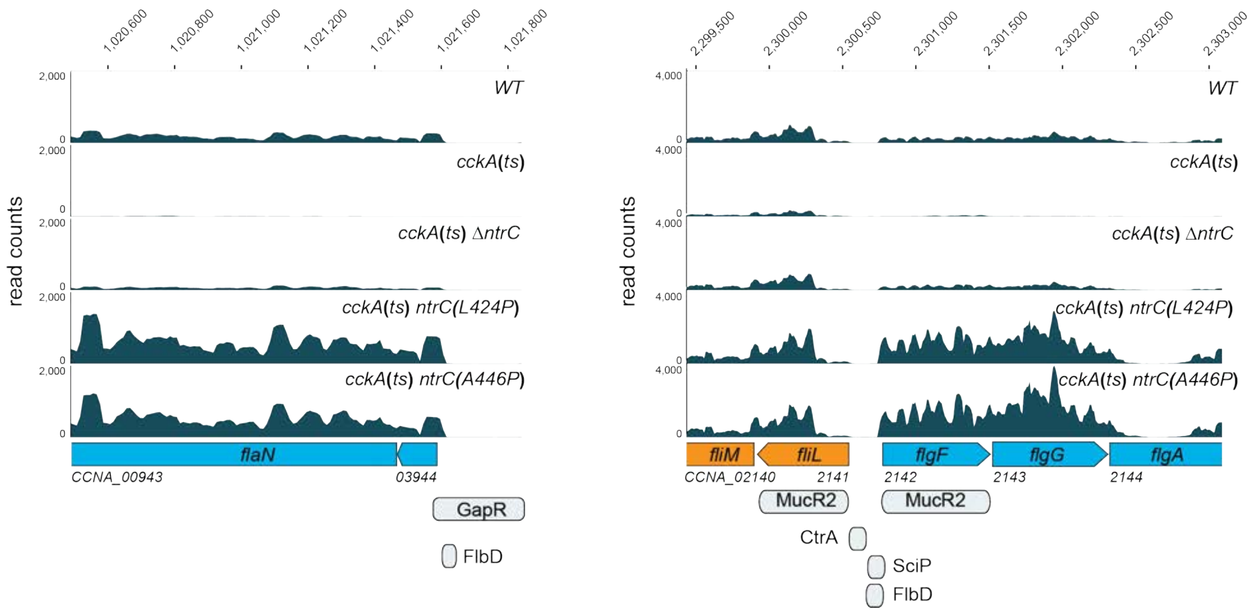

Figure S7. Mapped RNA sequencing read depth for select flagellar gene regions in wild type (WT), *cckA*(ts), *cckA*(ts) Δ*ntrC*, *cckA*(ts) *ntrC*(L424P), and *cckA*(ts) *ntrC*(A446P) strains. Class II flagellar genes are indicated in orange, and Class III flagellar genes are indicated in cyan. Locus numbers are indicated below the genes. Chromosome positions in the NA1000 genome (genbank CP001340) are indicated at the top. Binding sites for GapR, MucR, CtrA, SciP, and FlbD are also shown, highlighting their genomic positions relative to transcriptional activity in each strain.

References

1. Hartmann R, van Teeseling MCF, Thanbichler M, Drescher K. BacStalk: A comprehensive and interactive image analysis software tool for bacterial cell biology. Mol Microbiol. 2020;114(1):140-50. Epub 20200414. doi: 10.1111/mmi.14501. PubMed PMID: 32190923.
2. Vidangos NK, Heideker J, Lyubimov A, Lamers M, Huo Y, Pelton JG, et al. DNA recognition by a sigma(54) transcriptional activator from Aquifex aeolicus. J Mol Biol. 2014;426(21):3553-68. Epub 20140823. doi: 10.1016/j.jmb.2014.08.009. PubMed PMID: 25158097; PubMed Central PMCID: PMCPMC4188747.
3. Schrödinger L, DeLano W. PyMOL 2020. Available from: http://www.pymol.org/pymol.
